# Pattern-triggered immunity in blue and white seed cultivars of *Papaver somniferum*

**DOI:** 10.1093/aobpla/plaf055

**Published:** 2025-10-06

**Authors:** Jhonny Stalyn Hernández Orozco, Oksana Iakovenko, Adam Zeiner, Marie Hronková, Jiří Kubásek, Bára Kučerová, Iveta Vachová, Serban Pop, Natálie Hradecká, Petr Maršík, Markéta Macho, Pavla Fojtíková, Andrea Rychlá, Ondřej Hejna, Ivan Kulich, Michael Wrzaczek, Martin Janda

**Affiliations:** Department of Experimental Plant Biology, Faculty of Science, University of South Bohemia in České Budějovice, Branišovská 1645/31a, České Budějovice 370 05, Czech Republic; Department of Experimental Plant Biology, Faculty of Science, University of South Bohemia in České Budějovice, Branišovská 1645/31a, České Budějovice 370 05, Czech Republic; Department of Experimental Plant Biology, Faculty of Science, University of South Bohemia in České Budějovice, Branišovská 1645/31a, České Budějovice 370 05, Czech Republic; Institute of Plant Molecular Biology, Biology Centre, Czech Academy of Sciences, Branišovská 1160/31, České Budějovice 370 05, Czech Republic; Department of Experimental Plant Biology, Faculty of Science, University of South Bohemia in České Budějovice, Branišovská 1645/31a, České Budějovice 370 05, Czech Republic; Department of Experimental Plant Biology, Faculty of Science, University of South Bohemia in České Budějovice, Branišovská 1645/31a, České Budějovice 370 05, Czech Republic; Department of Experimental Plant Biology, Faculty of Science, University of South Bohemia in České Budějovice, Branišovská 1645/31a, České Budějovice 370 05, Czech Republic; Department of Experimental Plant Biology, Faculty of Science, University of South Bohemia in České Budějovice, Branišovská 1645/31a, České Budějovice 370 05, Czech Republic; Department of Experimental Plant Biology, Faculty of Science, University of South Bohemia in České Budějovice, Branišovská 1645/31a, České Budějovice 370 05, Czech Republic; Department of Experimental Plant Biology, Faculty of Science, University of South Bohemia in České Budějovice, Branišovská 1645/31a, České Budějovice 370 05, Czech Republic; Laboratory of Plant Biotechnologies, Institute of Experimental Botany, Czech Academy of Sciences, Rozvojová 263, Lysolaje, Praha 6 165 02, Czech Republic; Department of Food Science, Faculty of Agrobiology, Food and Natural Resources, Czech University of Life Sciences Prague, Kamýcká 129, Suchdol, Praha 6 165 00, Czech Republic; Department of Experimental Plant Biology, Faculty of Science, University of South Bohemia in České Budějovice, Branišovská 1645/31a, České Budějovice 370 05, Czech Republic; Department of Chemistry, Faculty of Science, University of South Bohemia in České Budějovice, Branišovská 1645/31a, České Budějovice 370 05, Czech Republic; OSEVA vývoj a výzkum s.r.o, Výzkumný ústav olejnin Opava, Purkyňova 10, Opava 764 01, Czech Republic; Department of Genetics and Biotechnologies, Faculty of Agriculture and Technology, University of South Bohemia in České Budějovice, Branišovská 1645/31a, České Budějovice 370 05, Czech Republic; Institute of Plant Molecular Biology, Biology Centre, Czech Academy of Sciences, Branišovská 1160/31, České Budějovice 370 05, Czech Republic; Department of Experimental Plant Biology, Faculty of Science, University of South Bohemia in České Budějovice, Branišovská 1645/31a, České Budějovice 370 05, Czech Republic; Institute of Plant Molecular Biology, Biology Centre, Czech Academy of Sciences, Branišovská 1160/31, České Budějovice 370 05, Czech Republic; Department of Experimental Plant Biology, Faculty of Science, University of South Bohemia in České Budějovice, Branišovská 1645/31a, České Budějovice 370 05, Czech Republic; Evolution & Diversity

**Keywords:** breadseed poppy, PAMP, DAMP, reactive oxygen species, MAPK, gene expression, ethylene, callose, wounding, salicylic acid

## Abstract

*Papaver somniferum* (poppy) is a traditional ingredient in Central and Eastern European cuisine and an important oilseed crop of the region. Since the main threat to stable poppy yield is pathogen infection, a detailed understanding of its defence mechanism is essential. The first robust layer of plant immunity, which plays a crucial role in combating pathogens, is pattern-triggered immunity (PTI). Here, we provide the first comprehensive insights into PTI in poppy. We selected four poppy varieties used in the food industry and investigated their response to various previously described peptide elicitors. Among all tested peptides, flg22 induced the most robust reactive oxygen species (ROS) burst, as well as triggering putative mitogen-activated protein kinase phosphorylation and seedling growth inhibition in all selected cultivars. We identified *PsWRKY22* and *PsPR2* as candidate marker genes suitable for monitoring poppy PTI responses. The tested poppy cultivars have low levels of salicylic acid. Callose accumulation was triggered by wounding but not by flg22. When studying PTI in plants, wounding is a challenge that needs to be considered, as it can obscure potential PTI responses. Our findings highlight conserved aspects of poppy immunity and the challenges of studying its PTI. The established pipeline facilitates improving our understanding of poppy immunity and has the potential for widespread application in breeding and improving selection for broad-spectrum disease resistance provided by enhanced PTI.

## Teaser

This study reveals how poppy plants activate immune defences against pathogens, focusing on pattern-triggered immunity (PTI). By testing four food-grade poppy varieties on the response to known immune-triggering peptides, we found that the peptide flg22 consistently activated robust immune responses, including oxidative bursts and growth inhibition. We identified two poppy genes, PsWRKY22 and PsPR2, as reliable markers of PTI. Interestingly, poppy plants showed low levels of salicylic acid and did not accumulate callose in response to flg22, highlighting unique aspects of poppy immunity. The findings offer a valuable framework for breeding poppy varieties with improved disease resistance.

## Introduction

Poppy cultivation has a long-standing history, with records dating back to 4000 Bc when the ancient Sumerians cultivated it. Poppy was called *Hul Gil* (‘flower of joy’) (museum.2025.gov 2025). Due to the presence of more than 80 alkaloids identified in *Papaver somniferum* (www.2025.europa.eu 2025), poppy remains important as both a medicinal and pharmaceutical crop (Beaudoin and Facchini 2014, Singh et al. 2019). It is also a major oilseed crop in Central and Eastern Europe (Neugschwandtner et al. 2023). The oil content of poppy seeds is around 40%–50%, with the oil being rich in vitamin E and minerals (Melo et al. 2022). The Czech Republic is the world's leading producer and exporter of poppy seeds per capita, contributing $88 million in exports in 2007 (Prochazka and Smutka 2012). Based on the Czech Statistical Office, in 2024, breadseed poppy covered 36 611 hectares in the Czech Republic, which is almost double compared to the area used for potatoes (22 747 ha), and the export in 2021 was worth around 60 million € (csu.2021.cz 2025).

According to United Nations estimates, up to 40% of global crop production is lost to pests annually, with diseases costing an estimated $220 billion, and invasive insects adding another $70 billion (www.fao.org). Like any other crop, poppy is vulnerable to pathogens and pests (Bailey et al. 2000, Thangavel et al. 2018). Breadseed poppy, bred for its low alkaloid content, has reduced natural defence capabilities. Consequently, significant diseases include leaf blight, caused by the fungal pathogen *Pleospora papaveracea* (O’Neill et al. 2000), and poppy downy mildew, caused by the oomycete *Peronospora arborescens* (Landa et al. 2007), both of which can devastate entire fields. Bacterial diseases such as bacterial blight, caused by *Xanthomonas campestris* pv. *Papavericola* (Gingeras et al. 1978) and bacterial stem rot, caused by *Erwinia carotovora* (Alam et al. 2014), also threaten poppy plants.

Understanding plant immunity is crucial for the mitigation of the losses caused by biotic stress. In the past thirty years, research on plant immunity, particularly in model plants like *Arabidopsis thaliana*, has provided invaluable insights into molecular mechanisms behind these processes. A key aspect of plant immunity is the recognition of pathogens. Plants detect two main types of pathogen molecules: (i) pathogen-associated molecular patterns (PAMPs) and (ii) effectors. PAMPs are recognized by pattern recognition receptors (PRRs), mainly located on the plasma membrane, while effectors are typically detected in the cytosol by receptor-like kinases (Jones and Dangl 2006). PAMP recognition triggers the first layer of plant immunity, known as pattern-triggered immunity (PTI). PAMPs are conserved molecules essential to pathogens and are chemically distinct from the hosts’ own. They are broad groups of molecules ranging from peptides (e.g. flagellin, elf18) and sugars (e.g. chitin) to short-chain fatty acids (Bigeard et al. 2015, Boutrot and Zipfel 2017). PTI can also be triggered by damage-associated molecular patterns (DAMPs) derived from plant components such as extracellular ATP, cell wall fragments, or peptides produced under stress (Tanaka and Heil 2021).

One of the best-studied ligand-receptor pairs involved in PTI is flg22, a 22-amino acid epitope of bacterial flagellin, and its receptor FLAGELLIN SENSING 2 (FLS2) (Tena 2019). Research in *Arabidopsis thaliana* has revealed the molecular mechanism of flg22 binding to FLS2, including the involvement of the co-receptor BAK1 (Sun et al. 2013). This interaction triggers several typical defence responses, such as transient Ca^2+^ spikes (Chi et al. 2021), apoplast alkalisation (Felix et al. 1999), reactive oxygen species (ROS) bursts (Smith and Heese 2014), mitogen-activated protein kinases (MAPK) cascade activation (Tian et al. 2021), increased callose deposition (Ellinger and Voigt 2014), dynamic transcriptional changes (Zipfel et al. 2004, Bjornson et al. 2021), and changes in phytohormone levels, which include salicylic acid (Tsuda et al. 2008) and ethylene (Felix et al. 1999). Responses to flg22 have been observed in numerous plant species, including members of the *Solanaceae*, *Brassicaceae* (Nguyen et al. 2010, Lloyd et al. 2014), and *Poaceae* (Takai et al. 2008). Plant breeding and biotechnology have shown to benefit from a more detailed understanding of PTI. For example, the transfer of the PRR EF-Tu receptor (EFR) from *Arabidopsis*, which recognizes a peptide from the bacterial elongation factor elf18, to tomato has significantly increased resistance to bacterial infections (Lacombe et al. 2010). Importantly, no systematic study focusing on PAMPs or DAMPs, including flg22, has been conducted on breadseed poppy (*Papaver somniferum*) so far.

This study aims to establish, optimise, and present a methodology suitable for the analysis of the PTI responses in poppy. We observed that flg22 is the most potent of the tested PAMP- and DAMP-derived peptides; therefore, we used flg22 as a representative PTI elicitor. Our results provide the first picture of poppy immune responses, establishing a basis for PTI research that can guide poppy breeding efforts towards increased resistance to pathogens in the future.

## Materials and methods

### Plant material

We used four *Papaver somniferum L.* cultivars in this study. The cultivars were provided by OSEVA PRO s.r.o (Opava, Czech Republic): Gerlach (ID nr. 15O0800148), Orel (ID nr. 15O0800187), Opex (ID nr. 15O0800203), and Olaf (new cultivar, no ID nr. at the time of writing the manuscript). Plants were grown in two distinct styles for the experiments: *in vitro* (sterile conditions; seedlings) and in soil (non-sterile conditions; adult plants). For both cultivation styles, seedlings were sterilized using 30% (v/v) sodium hypochlorite solution for 6–10 min and washed four times using sterile distilled water (*in vitro* conditions) or distilled water (in soil conditions). Sterilized seeds were stratified (4°C) in the dark in water for 1–5 days prior to sowing. Similar growing conditions were used for *Arabidopsis thaliana* plants. Potato plants were grown in soil in the greenhouse under uncontrolled light conditions, only the maximum temperature being controlled to be under 26°C. 3–6 weeks old potato plants were used for the experiments. Potatoes were used as a control of elicitor functionality.

*In soil* conditions, seeds were sown in perlite within a hydroponic system and watered with hydroponic nutrient solution containing Jungle Garden Base 0.1% (v/v) and Jungle Garden G1 0.25% (v/v) (JUNGLE Indabox, Czech Republic). After 6–9 days, seedlings were transferred into a peat pellet (Jiffy 7, Bohemiaseed, Czech Republic) (Supplementary Figure S1). The plants were watered with distilled water approximately once a week. Plants were grown for 5–6 weeks in a Phytotron (Photon Systems Instruments, Czech Republic) under a short-day photoperiod (10 h day/14 h night regime), light intensity of 120 μmol·m^−2^·s^−1^, at a temperature of 22°C/18°C day/night, and ∼60% relative humidity.

*In vitro* conditions, seeds were sown on solid Murashige and Skoog medium (MS) with vitamins (Duchefa, Netherlands) containing 1% (w/v) sucrose and 1.2% (w/v) Gelrite (Duchefa, Netherlands), pH was adjusted to 5.6–5.7 (Supplementary Figure S2). The seedlings grew on solid media for 2–5 days under a 16 h day/8 h night regime, light intensity of 110–140 μmol·m^−2^·s^−1^, at 23–25°C. Seedlings were then moved to a 24-well plate with 400 μl of MS liquid medium with vitamins and 1% (w/v) sucrose, and placed under the same growing conditions as on solid media. *In vitro* seedlings were used for the ROS analysis, seedling growth inhibition assay (for this assay, sucrose-free liquid MS medium was also used), and callose measurement (further details in dedicated sections).

### Luminol-based assay for measurement of reactive oxygen species production

The ROS production was determined using the luminol-based assay previously described in (Janda et al. 2019).

#### Reactive oxygen species measurement in leaves from adult poppy plants grown on soil (in soil conditions)

Leaf discs (4 mm in diameter) of 5–6-week-old poppy plants (leaves were used from at least three independent plants per one biological replicate) were incubated in 100 μl of distilled water into a white 96-well plate, in the dark at room temperature, overnight. The water was then replaced with the solution containing 200 μM luminol (Serva, 28085.02), 20 μg/ml horseradish peroxidase (HRP, Apollo Scientific, Aposbitp1327), and a particular elicitor (concentration of used elicitors is specified in the results section and figure legends): flg22 (EZBiolab; QRLSTGSRINSAKDDAAGLQIA; Chinchilla et al. 2006), *Xcc*Flg22 (Nzytech; QRLSSGLRINSAKDDAAGLAIS; this study), flgII-28 (Chinese Peptide; ESTNILQRMRELAVQSRNDSNSATDREA; Moroz and Tanaka 2020), elf18 [EZBiolab; Ac-SKEKFERTKPHVNVGTIG; Kunze et al. 2004), *At*Pep1 (Chinese Peptide; ATKVKAKQRGKEKVSSGRPGQHN; Ortiz-Morea et al. 2016), csp22 (Chinese Peptide; AVGTVKWFNAEKGFGFITPDDG; Trinh et al. 2024), and Pep13 (Chinese Peptide; VWNQPVRGFKVYE.; Nietzschmann et al. 2019). The elicitors were used in order to induce the production of ROS, which was monitored as luminescence intensity correlating with the concentration of H_2_O_2_ and described as relative luminescence unit, as the area under the curve from the 4th to 24th min (total photon counts), or as photon counts in the peak of the curve (photon counts [max value]). For measuring luminescence, a Tecan SparkCyto 300 (Tecan, Switzerland) luminometer was used.

#### Reactive oxygen species measurement in seedlings grown *in vitro*

Five-day-old seedlings grown on the solid medium in sterile conditions were moved to 24-well plates containing MS liquid medium (described above) for 24 h. Then, rinsed seedlings were individually moved into a white bottom 96-well plate with 200 μl distilled water and kept in the dark for at least 16 h. After that, water was replaced by 200 μl of 5 μM flg22, HRP (20 μg/ml), luminol (200 μM), and distilled water. Seedlings treated with distilled water instead of flg22 were used as a control. One seedling represents one independent sample within one biological replicate. One biological replicate contained 12 seedlings.

### Measurement of Reactive oxygen species in poppy root apoplast using Amplex Red

The ROS production in roots was determined using the Amplex Red Reagent (Invitrogen, A12222) method previously described in (Kulich et al. 2025). An FV1000 Olympus Confocal Microscope with a UPlanSApo 10×/0.40 objective (wavelengths: excitation 559 nm; emission 583 nm) was used for imaging. For experiments, roots of 3–4-day-old poppy seedlings grown on 1/2 MS medium with vitamins, 1% (w/v) plant agar, and 1% (w/v) sucrose were used. Poppy seedlings were placed on a glass microscope slide in a drop of 1/2 MS medium and were left to rest for ∼2 min, after which it was washed off with mock solution, followed by the addition of a 1 μM flg22 solution replacing the original medium. The drop in fluorescence intensity over time after medium replacement was observed, then compared to the control sample. This stain predominantly localizes to the apoplast close to the elongation zone and exhibits a red fluorescence halo upon oxidation to resorufin in the presence of H_2_O_2_. Images were captured every 3 seconds. Fiji ImageJ (version 8) was used for the image analysis. Intensity of stain on the ROI localized close to the apoplast of the elongation zone was measured. The mean grey value at *t* = 0 s is subtracted from all following values. The data was not normalized. One seedling represents one independent sample; at least four seedlings for each variant were used within one biological repeat.

### Mitogen-activated protein kinase activation assay

For analysis of the putative MAPK phosphorylation in poppy, we used leaves from 5- to 6-week-old plants. We used two styles of 5 μM flg22 treatments:

#### Leaf infiltration with needleless syringes

Non-treated leaves, parts of leaves infiltrated with distilled H_2_O, or parts of leaves infiltrated with 5 μM flg22 were collected 15 and 30 min after the infiltration and frozen in liquid nitrogen. Leaves from at least three plants were used as an independent sample.

#### Leaf disc treatment

Leaf discs (4 mm) were either immediately frozen in liquid nitrogen (steady negative control) or incubated at least 16 h in distilled water under continuous dark conditions and then treated by replacing the water either with fresh distilled water or with a 5 μM flg22 solution. The treatment was performed for 15 and 30 min. After that, the leaf discs were frozen in liquid nitrogen. For obtaining discs, leaves from at least three plants were used, which represent one independent sample for each variant.

Collected samples were homogenized in liquid nitrogen using a mortar and pestle. Proteins were extracted (50 mM HEPES, pH 7.5; 75 mM NaCl; 1 mM EGTA; 1 mM MgCl_2_; 1 mM NaF; 10% (v/v) glycerol; 1 mM DTT; cOmplete, EDTA-free Protease Inhibitor Cocktail (Roche, 11873580001) and Pierce Phosphatase Inhibitor Mini Tablets (Thermo Scientific, A32957)) and quantified by Bradford assay (Bradford 1976). 15 μg of total protein was separated by 12% SDS-PAGE and transferred to a PVDF membrane (Immun-Blot Low Fluorescence PVDF Membrane, BioRad, 1620264). The membrane was blocked with 5% (w/v) bovine serum albumin (BSA) in Tris-buffered-saline (TBS)-0.1% (v/v) Tween-20 (T) (1 h at room temperature, or overnight at 4°C), incubated with primary antibody (Phospho-p44/42 MAPK (Erk1/2) (Thr202/Tyr204) Antibody, Cell Signalling Technology, #9101; diluted 1:1000 in 1% (w/v) BSA in TBS-T, overnight at 4°C), washed in TBS-T (5 times 5 min at room temperature), incubated with secondary antibody (StarBright™ Blue 520 Goat Anti-Rabbit IgG, BioRad, #12005870; diluted 1:2500 in 1% (w/v) BSA in TBS-T, 30 min at room temperature), and finally washed in TBS (5 times 5 min at room temperature). The fluorescent antibody was detected with a documentation unit ChemiDoc (BioRad) with the provided protocol for the detection of StarBright Blue 520. The loading control is represented as Coomassie dye (0.25% (w/v) Coomassie Brilliant Blue R-250, 45% (v/v) methanol, 10% (v/v) glacial acetic acid) stained, and destained (45% (v/v) methanol, 10% (v/v) glacial acetic acid) membrane, which was documented with already declared documentation unit. Analysis was repeated two times with similar result. Final images were analysed with Image Lab (BioRad, 6.0.1).

### Seedling growth inhibition assay

Two- to 5-day-old poppy (or Arabidopsis) seedlings grown on solid medium of full MS with vitamins, containing 1% (w/v) sucrose, (*in vitro* conditions) were moved to 24-well plates containing 500 μl MS with vitamins liquid media with or without 1% (w/v) sucrose (Duchefa, Netherlands). These were supplemented by either 5 μM flg22 or sterile H_2_O. One to three seedlings were placed into each well. Media ±flg22 were refreshed after 3 days in experiments using MS media with sucrose. In those without sucrose, we did not refresh the media. Seedlings were cultivated for 5–7 days. Thus, 10–12 days old seedlings were harvested for analysis. The seedlings were weighed individually. Each seedling represents an independent sample for each variant. At least eight seedlings were used for each variant within each biological repeat.

### Gene expression analysis

For the analysis of gene expression in poppy, we used a leaf disc treatment approach (similar to the ROS assay method). Discs were obtained from leaves of 5–6-week-old plants. Leaves from at least three plants were used for each variant as an independent sample. Discs were either immediately frozen after collection (steady negative control) or incubated overnight in distilled water and treated with fresh distilled water or with a water solution containing 5 μM flg22 for the corresponding time (as indicated). After that, the leaf discs were frozen in liquid nitrogen. Three to four independent replicates for each treatment and each time point were prepared.

For RNA extraction, between 60 and 130 mg of fresh weight was used. Samples were homogenized in tubes with 1.3 mm silica beads, using a FastPrep-24 instrument (MP Biomedicals, USA). Total RNA was extracted from leaves or discs of *P. somniferum* using the FavorPrep Total RNA Isolation Kit (FAVORGEN Biotech Corp.) according to the manufacturer’s instructions. Part of the RNA samples were treated with the DNA-free Kit, DNase Treatment and Removal (Invitrogen, AM1906) to eliminate genomic DNA contamination, and 1 μg of pure total RNA was used for the synthesis of cDNA by the High-Capacity cDNA Reverse Transcription Kit (Applied Biosystems, 4368814) according to the manufacturer’s instructions. RT-qPCR was performed with a qTower3 Real-time PCR detection system (Analytic Jena) using HOT FIREPol Eva Green qPCR Mix Plus ROX (SOLIS BIODYNE). 4 μl (1:20 diluted) of the RT product in a final reaction volume of 20 μl was used. The following PCR program was used throughout the study: 95°C for 12 min, followed by 40 cycles of 95°C for 15 s, 55°C for 20 s, and 72°C for 15 s. Two technical replicates were set up for each cDNA template. Data were normalized to the reference gene Actin and to the transcript level relative to the non-treated or mock samples (as indicated in figure legends) by the comparative CT method (Livak and Schmittgen 2001). The primers used in qRT-PCR are listed in Supplementary Table S1.

Expression values were normalized to the mock samples (water treated) or to the steady negative control (indicated in the figure legend), which were set to 1. It means that all values were divided by the mean from either mock samples or steady negative control samples. Thus. expression levels in treated samples are presented relative to the corresponding control (indicated in figure legend).

### Callose accumulation analysis

Treatment with flg22 for callose accumulation analysis in poppy plants was done in three ways: using 8-day-old seedlings grown *in vitro*, leaf discs, and whole leaves from 4–5-week-old plants.

Seedlings grown in the same way as for the growth inhibition assay were transferred for 72 h to fresh MS medium with vitamins, containing 1% (w/v) sucrose, (mock) or to the same medium supplemented with 5 μM flg22. At least 10 seedlings were used for each variant within one biological replicate.

For the leaf infiltration, 4–6 plants had three leaves at the same developmental stage were infiltrated with 5 μM flg22 solution or distillate water (mock). 72 h after treatment, these leaves and twelve additional leaves from four non-treated plants (three each) were collected for further staining procedure. One leaf represents an independent sample. Leaves from at least three plants were used for each variant within one biological replicate.

In the leaf disc treatment, four poppy plants had 4 mm discs cut from three leaves and individually placed into the wells of a 96-well plate, to which 100 μl of distilled water was added. After overnight incubation in the dark, at room temperature, the solution was replaced by 5 μM flg22 or fresh distilled water (mock), and further incubated for 12 hours. Freshly cut discs were collected immediately before staining to serve as a control. One disc represents an independent sample. Leaves from at least three plants were used for cutting discs for each variant within one biological replicate.

After treatment, plant material was fixed in 96% ethanol: glacial acetic acid (3:1, v/v), the solution was replaced several times until plant tissue was decolourized. After that, it was rehydrated and sequentially incubated in 70/50/30% (v/v) ethanol and distilled water for 1 hour in each solution. Finally, fixed tissue was stained with 0.01% (w/v) aniline blue in 150 mM K_2_HPO_4_ (pH 9.5) overnight.

Callose deposition was observed using DAPI channel (wavelengths: excitation 359 nm/emission 455 nm), on a BX63 Olympus widefield fluorescence microscope, using a 4X objective Olympus UPlanXApo 4X/0.16. The ratio of the area of all callose spots to the area of the whole leaf or disc was calculated using Labkit in Fiji ImageJ v8 software (Schindelin et al. 2012).

### Poppy FLS2 receptor and flg22 binding analysis

A FLS2 homologous protein of *P. somniferum L.* was identified using *A. thaliana* FLS2 protein sequence (AT5G46330.1) as a Blastp query (Altschul et al. 1990). Blastp was set up to query FLS2 only in *Papaver somniferum L*. (NCBI ID ASM357369v1), the high-scoring subject protein LRR receptor-like serine/threonine-protein kinase FLS2 (NCBI ID XP_026454075.1) was selected for subsequent modelling.

The amino acid sequence of putative *Ps*FLS2 was used for *in silico* prediction of 3D structure using the AlphaFold2 server version 2.3.0 (Jumper et al. 2021). In the *Ps*FLS2 modelled structure, only the extracellular domain (LRRs region) was conserved.

*Xcc*flg22 peptide sequence was obtained through Blastp using as query sequence *Pseudomonas syringae* pv*. tomato str. DC3000* flagellin (NCBI ID AAO55467.1). Hit protein sequence in *Xanthomonas campestris* pv. *Campestris* named as flagellin (NCBI ID MEB1151700.1) was used. To obtain *Xc*flg22 3D structure, the flg22 peptide structure from Sun et al. (2013) (RCSB PDB ID 4MN8) was used as a template and modelling was performed in PyMOL (Molecular Graphics System, Version 3.1 Schrödinger, LLC.).

*Ps*FLS2 and *Xcc*flg22 PDB files were treated using OpenBabelGUI 3.1.1 (O’Boyle et al. 2011) to add charges in the residues and convert PDB to PBDQT files. The PBDQT files were used for the molecular docking process through PyRx- Python Prescription 0.8 Virtual Screening Tool (Dallakyan and Olson 2015) a visual interface of AutoDock4 v4.2.6 program (Morris et al. 2009). The Autodock wizard was set up for docking, with a modified grid to cover the entire FLS2 section. Grid area was determined by an alignment between the *Ps*FLS2 (XP_026454075.1) and *At*FLS2 (AT5G46330.1) protein sequences using MEGA11: Molecular Evolutionary Genetics Analysis version 11.0.10 (Tamura et al. 2021). The alignment was performed with the MUSCLE algorithm (with default settings) (Supplementary Figure S7). Potential *Ps*FLS2 residues that could interact with *Xcc*flg22 or flg22 were selected using the list of residues in *At*FLS2 that interact with flg22 reported by (Sun et al. 2013).

In *At*FLS2 docking, 3D structure (RCSB PDB accession number 4MNA) was prepared by removing water and other molecules using PyMOL, and the resulting PBD file was prepared in the same manner as *Ps*FLS2. After the docking process, the ligand with the lowest binding energy (B.A.) was selected and for visualization and imaging of the complexes, an open-source web-based toolkit Open Mol*Viewer (Sehnal et al. 2021) was used. B.A. was used to calculate *in silico* Kd (equilibrium dissociation constant) using the Gibbs-Helmholtz equation.

### Salicylic acid measurement

We analysed salicylic acid (SA) concentration as described previously (Leontovyčová et al. 2019, Pluhařová et al. 2019). Briefly, analysis of SA content was carried out in four biologically independent samples from each variant (treatment, cultivar), with every sample containing 100–200 mg fresh weight leaves from 5- to 6-week-old poppy plants. Material from at least three plants was collected and considered as one independent sample. Samples were homogenized in tubes with 1.3 mm silica beads using a FastPrep-24 instrument (MP Biomedicals, USA). For the extraction of soluble fraction, a methanol/H_2_O/formic acid (15:4:1, v:v:v) mixture supplemented with stable-isotope-labelled ^13^C_6_-SA internal standards was used. Extracts were subjected to solid phase extraction using Oasis MCX cartridges (Waters Co., Milford, MA, United States) and eluted with methanol. The eluate was dried and dissolved in 15% (v/v) acetonitrile/water directly before the analysis. Quantification was performed on an Ultimate 3000 high-performance liquid chromatograph (UHPLC, Dionex; Thermo Fisher Scientific, Waltham, MA, United States) coupled to an IMPACT II Q-TOF ultra-high resolution and high-mass-accuracy mass spectrometer (HRAM-MS; Bruker Daltonik, Bremen, Germany). Separation was carried out using an Acclaim RSLC 120 C18 column (2.2 m, 2.1 × 100 mm; Thermo Fisher Scientific, Waltham, MA, United States) tempered at 35°C and mobile phase consisting of 0.1% (v/v) formic acid (solvent A) in methanol (solvent B) by 16-min gradient elution at a flow rate of 0.3 ml/min. The linear gradient started at 1% solvent B (0–1 min), increased to 39% at 3 min, then to 60% at 7 min, and finally to 99.9% at 8–11 min, followed by equilibrium at the initial value of 1% B from 12 to 16 min. Injection volume was 5 μl. The full-scan data were recorded in negative electrospray ionization (ESI^−^) mode. The signals of SA and internal standard (^13^C_6_-SA) were monitored as deprotonated molecular ions [M-H]^−^ (137.0239 *m/z* and 143.0440 *m/z*, respectively). Spectra were acquired with a mass resolution of >60 000 and a scan rate of 0.5 Hz.

### Ethylene measurement

The analysis of ethylene production was adapted from (Felix et al. 1999). In brief, six-fully expanded poppy or Arabidopsis leaves from 5- to 6-week-old plants cultivated in soil were either untreated (control) or infiltrated with distilled water (mock) or with 5 μM flg22 and put into 20 ml glass vials containing 10 ml of distilled water. Vials were closed with silicone/PTFE septa and leaves were incubated at room temperature in the dark for four hours. Ethylene accumulated in the free air space was measured using gas chromatography. Ethylene was separated from atmospheric methane and other volatiles using PLOT column (Rt-Q-BOND, RESTEK, 30 m × 0.25 mm ID and 8.4 μm film thickness) under temperature of 38°C and column flow of 2 ml min^−1^ and detected by flame ionization detector. At least three leaves from three plants represent one independent sample.

### Statistical data analysis and creation of graphs

For statistical data analysis and graph creation, either MS Excel (Microsoft 365) or GraphPad Prism 10.0.0 (GraphPad Software, Boston, Massachusetts USA) were used. Detailed information about statistical analysis is available in the respective figure legends. Immunoblot figures were prepared using Inkscape (Inkscape Developers, 1.3.2), and finalized in PowerPoint (Microsoft 365), as with all other figures.

## Results

For our experiments, we selected four poppy cultivars: Gerlach (spring blue seed), Olaf (overwintering blue seed), Orel (spring white seed), and Opex (spring blue seed). Standard growing conditions were established using *in soil* peat pellets (Supplementary Figure S1) and *in vitro* sterile cultivation media (Supplementary Figure S2).

### Reactive oxygen species burst in *P. somniferum* leaves

**Figure 1.**
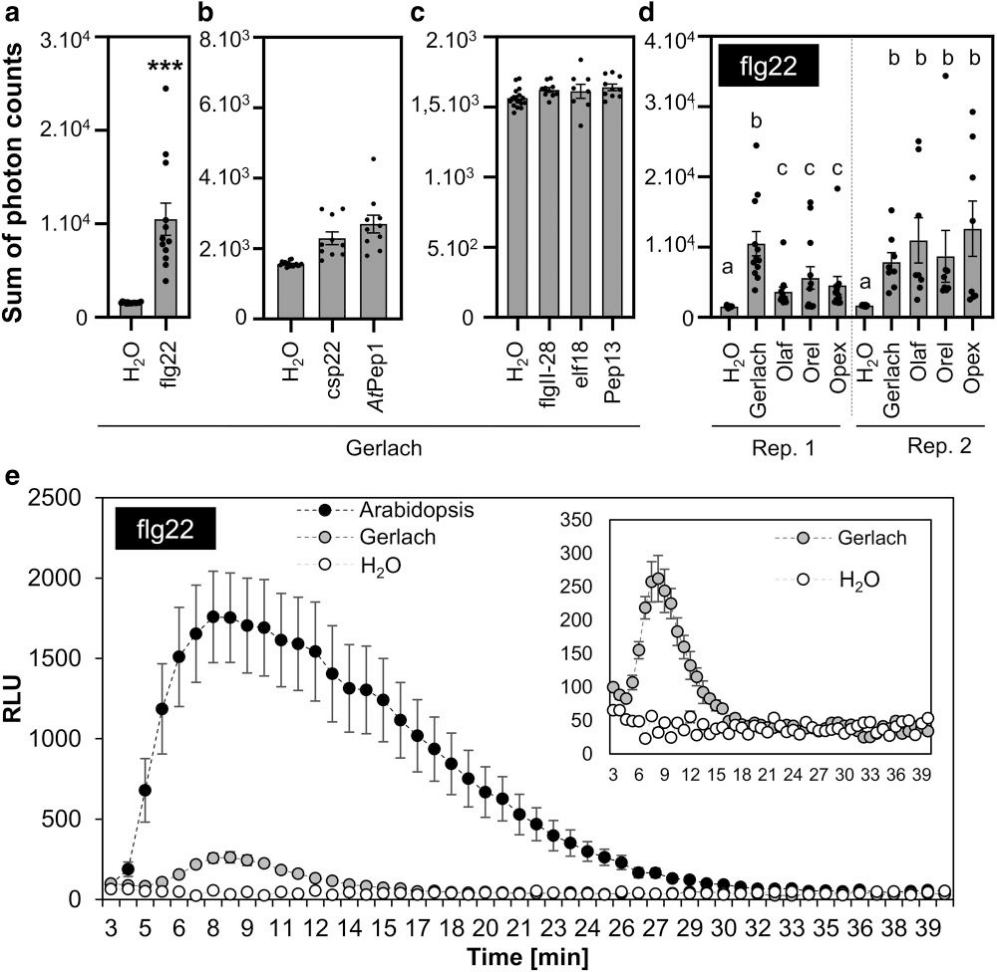
ROS burst in poppy after treatment with peptide elicitors. Discs were cut from 5- to 6-week-old poppy plants (a–e) or 5–6-week-old *A. thaliana* plants (e). (a–c) Leaf discs from the Gerlach cultivar were treated with 1µM: flg22, flgII-28, csp22, *At*Pep1, elf18, and pep13. (d) Discs from Gerlach, Olaf, Orel, and Opex cultivars were treated with 1 μM flg22. (e) Comparison of the response to 1 μM flg22 in Gerlach and *A. thaliana*. The data are presented as mean ± SEM; *n* = 8–16 discs in one biological experiment. The experiments were repeated in at least three biological repeats with similar results. Asterisks in Fig.1a indicate that the mean value is significantly different from the control conditions (two-tailed Student’s *t*-test, ***P < .001). Statistical differences between the samples within particular repeats (d) were assessed using a one-way ANOVA, with a Tukey honestly significant difference (HSD) multiple mean comparison *post hoc* test. Different letters indicate a significant difference, Tukey HSD, *P* < 0.01. Absence of letters in b and c indicates no statistically significant differences.

ROS production is a rapid PTI response. We measured ROS using a luminol-based assay in leaf discs in 96-well plates (Smith and Heese 2014). We treated poppy with five well-known peptide PAMPs (flg22, flgII-28, elf18, csp22, pep13) and one peptide DAMP (*At*Pep1). Among the tested peptides, flg22 from *Pseudomonas aeruginosa* (further referred to as flg22) triggered the most potent ROS burst in all cultivars (Fig. 1a–d, Supplementary Figures S3, S4), while *At*Pep1 and csp22 induced weak, but detectable, responses (Fig. 1b and Supplementary Figure S3a). The peptides elf18, pep13, and flgII-28 did not elicit any observable ROS response (Fig. 1c and Supplementary Figure S3a). To demonstrate the functionality of used peptides which did not trigger a robust response (*At*Pep1, csp22, elf18, pep13, and flgII-28), we measured ROS production after treatment with peptides in plant species (potato and *A. thaliana*) previously described in the literature to be susceptible (Supplementary Figure S5) (Moroz and Tanaka 2020). Flg22 consistently triggered ROS production across all cultivars (Fig. 1d, Supplementary Figures S3b and S4). The response was more intense in Arabidopsis compared to poppy, and a lower flg22 concentration was sufficient to saturate the response in Arabidopsis compared to poppy (Fig. 1e and Supplementary Figure S6).

### Flg22 and *Xcc*Flg22 interaction with Arabidopsis and putative *P. somniferum* FLS2

We identified a putative *PsFLS2* (NCBI ID XP_026454075.1) in the poppy genome based on the alignment of protein sequences with AtFLS2 (Supplementary Figure S7). The similarity between *At*FLS2 and *Ps*FLS2 based on Blastp was 50.35 and the query coverage 96% with E-value 0.0. We modelled *Ps*FLS2 structure (Supplementary Figure S8), and used it for molecular docking.

**Figure 2.**
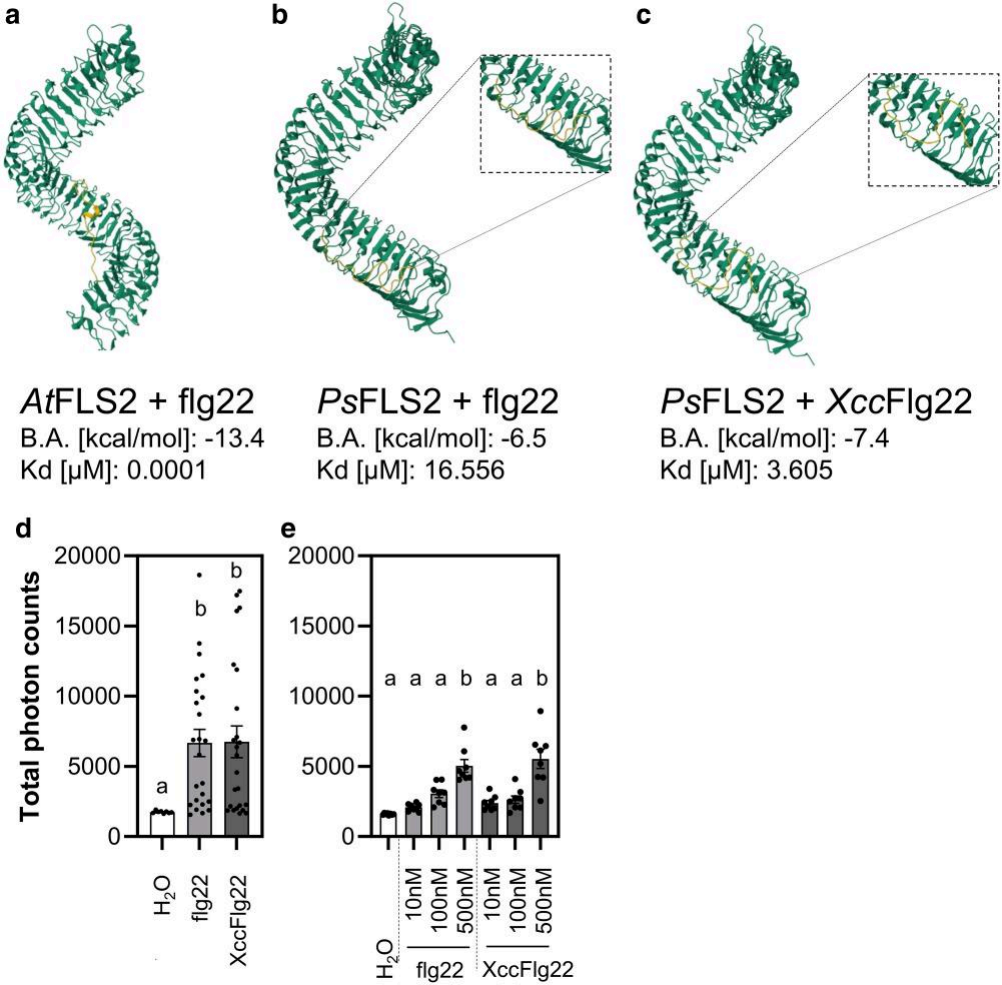
Response to flg22 originating from *Xanthomonas campestris* pv. *campestris*. (a) Binding of *Pseudomonas aeruginosa* flg22 (flg22) to *Arabidopsis thaliana* FLS2 (*At*FLS2). (b) Binding of *Pseudomonas aeruginosa* flg22 (flg22) to *Papaver somniferum* FLS2 (*Ps*FLS2). (c) Binding of *Xanthomonas campestris* pv. *campestris* flg22 (*Xcc*Flg22) to *Papaver somniferum* FLS2 (*Ps*FLS2). B.A.—Binding affinity; Kd (equilibrium dissociation constant) is estimated *in sillico*. (d and e) Discs were cut from 5- to 6-week-old Gerlach plants. Discs were treated with 1 μM flg22 or *Xcc*Flg22 (d) and 10–500 nM flg22 or *Xcc*Flg22 (e). The data are presented as mean ± SEM; *n* = 8–16 discs in one biological experiment. The experiment (d) was repeated in four biological repeats, and the experiment (e) in three biological repeats. Statistical differences between the samples (d, e) were assessed using a one-way ANOVA, with a Tukey honestly significant difference (HSD) multiple mean comparison *post hoc* test. Different letters indicate a significant difference, Tukey HSD, *P* < .01.

Molecular docking analysis showed a stronger binding affinity (Agu et al. 2023, Spassov 2024) of *At*FLS2 to flg22 (Fig. 2a) compared to *Ps*FLS2 to flg22 (Fig. 2b). We used binding affinity (B.A.) to calculate *in silico* equilibrium dissociation constant (Kd) representing the ligand concentration required to occupy 50% of the receptors (Hulme and Trevethick 2010). *In silico* calculated Kd values were 0.0001 μM for *At*FL2-flg22 and 16.556 μM for *Ps*FLS2-flg22, showing that for *At*FLS2 the concentration to reach Kd is roughly 1.10^5^ lower than the concentration needed for reaching Kd in *Ps*FLS2 using flg22 as a ligand (Supplementary Table S2).

The flg22 peptide (QRLSTGSRINSAKDDAAGLQIA), typically used in studies focused on plant immunity, is derived from *Pseudomonas aeruginosa* (Trinh et al. 2023). However, *P. aeruginosa* is not a typical poppy bacterial pathogen, so we examined a variant, *Xcc*Flg22 (QRLSSGLRINSAKDDAAGLAIS), from poppy pathogen *Xanthomonas campestris* pv. *Campestris*. Molecular docking studies showed that *Xcc*Flg22 had a higher binding affinity for *Ps*FLS2 (Fig. 2c and Supplementary Table S2), compared to flg22 and *Ps*FLS2 (Fig. 2b and Supplementary Table S2). However, no significant difference between flg22 and *Xcc*Flg22 was observed in their ROS-inducing effects in poppy (Fig. 2d and e, Supplementary Figures S9 and S10). We decided to continue further PTI response analyses in poppy using flg22 peptide from *P. aeruginosa*.

### Mitogen-activated protein kinase phosphorylation in *P. somniferum*

**Figure 3.**
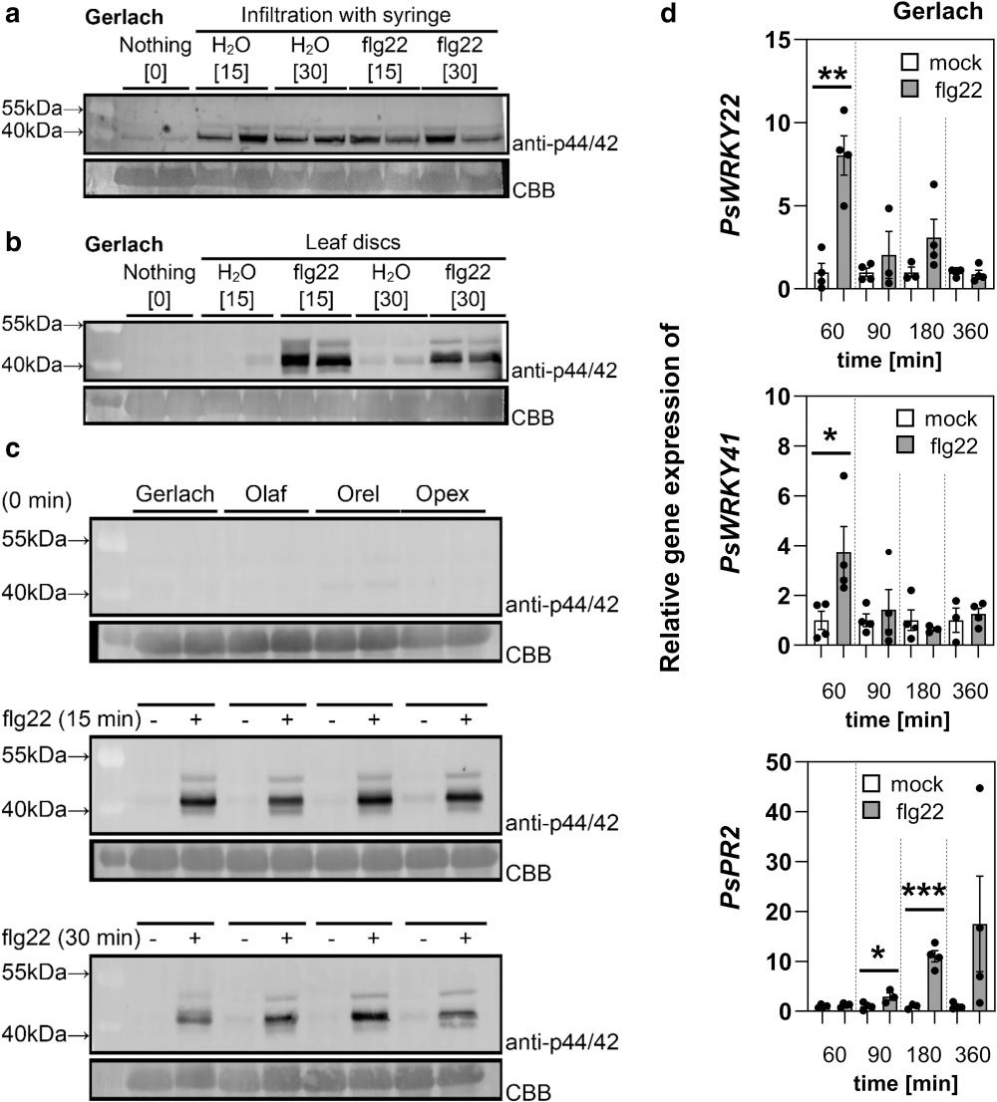
MAPK activation reflected by the abundance of pERK antibody recognized protein and gene transcription after treatment with flg22. The activation of the mitogen-activated protein kinases (MAPKs) was visualized by the western blot analysis using the pERK phospho-p44/42 MAP kinase antibody. Coomassie Brilliant Blue (CBB) staining of the membrane was used as a loading control. (a) Needleless syringe infiltration with 5 μM flg22 (or water) of leaves from 5- to 6-week-old poppy plants. (b) Treatment of leaf discs from 5- to 6-week-old Gerlach plants with 5 μM flg22 (or water). c) Treatment of leaf discs from 5- to 6-week-old poppy plants (four genotypes) with 5 μM flg22 (or water). (d) The leaf discs were cut from 5- to 6-week-old poppy and treated with 5 μM flg22 for 60, 90, 180, and 360 min. Expression values were normalized to the mock samples (water treated), which were set to 1. Expression levels in treated samples are presented relative to the corresponding control (mock at the same time point). The relative transcription for controls (water treated samples) was normalized to mean 1. The data are presented as mean ± SEM; *n* = 3–4. Asterisks indicate that the mean value is significantly different from the control conditions (two-tailed Student’s *t*-test, *P < 0.05, **P < 0.01, ***P < 0.001).

Activation of the mitogen-activated protein kinase (MAPK) cascade, like the ROS burst, is a fast response triggered by the recognition of flg22 in plants (Frey et al. 2014). We studied putative MAPK activation in poppy by using a pERK-antibody, which recognizes phosphorylation of the canonical TEY motif in MAPKs, as a proxy of MAPK pathway activation (Yi et al. 2015) at two time points (15 and 30 min) following treatment (Fig. 3a–c). Initially, we performed the needleless syringe infiltration using Gerlach as a model cultivar (Fig. 3a). As a result, we observed that wounding caused by infiltration of water also triggered putative MAPK activation at levels comparable to infiltration with flg22 (Fig. 3a). To overcome this problem, we used the same approach as for ROS burst measurement. Prior to the treatment, we incubated prepared leaf discs overnight in distilled water in the dark. Subsequently, we replaced the water with fresh water (mock) or with a solution containing flg22 without causing any mechanical stress to the leaf discs. We observed an increased abundance of the protein recognized (putative MAPKs) by the pERK antibody upon flg22 treatment in the Gerlach cultivar (Fig. 3b). As the incubation allowed us to overcome the activation caused by wounding, we used the leaf disc method to analyse all cultivars. In all of them, flg22 increased the abundance of pERK-recognized protein after 15 and 30 min (Fig. 3c, full blots in Supplementary Figure S11).

### Gene expression analysis of *P. somniferum*

We analysed gene expression changes after flg22 treatment to identify genes suitable for PTI monitoring in poppy. For this purpose, we used a leaf disc treatment similar to the ROS burst and MAPK assay. We designed primers for orthologs of the flg22 responsive genes described in Arabidopsis (*PsWRKY22, PsWRKY33* and *PsFRK1*) (Zou et al. 2018, Bjornson et al. 2021), for flg22 receptor (*PsFLS2*) (Chinchilla et al. 2006), and for *PR2* gene (*PsPR2*), whose expression is sensitive to biotic and abiotic stress, but known to also respond to flg22 (Liu et al. 2023). Additionally, we studied the expression of three published putative poppy genes whose transcription was increased upon treatment with bacterial endophyte *Microbacterium* sp. SMR1 (*PsCRK1*, *PsWRKY53*, *PsPRTS*) (Ray et al. 2021). We reannotated these three genes as the previously assigned names were based on a comparison with the annotated soybean genome. However, using the available poppy genome (Guo et al. 2018), primers designed for *PsCRK1* actually amplified *PsCRK35*; primers for *PsWRKY53* amplified *PsWRKY41*; and primers for *PsPRTS* amplified the gene encoding a thaumatin-like protein. Among all the tested genes, *PsWRKY22*, *PsWRKY41*, and *PsPR2* showed significantly enhanced transcript abundance following flg22 treatment in at least one analysed time point (Fig. 3d). *PsWRKY22* and *PsWRKY41* transcript levels were increased within 60 min and decreased over later time points (Fig. 3d), while *PsPR2* transcript abundance started to increase after 90 min and increased further 180 and 360 min after treatment (Fig. 3d). Other genes showed no significantly altered transcript levels after flg22 treatment (Supplementary Figure S12). However, we realized that using the discs for gene expression analyses is not ideal because we observed a significant effect on expression caused by cutting (Supplementary Figure S13). Although *PsWRKY22* exhibited clear induction of expression after flg22 treatment compared to mock samples, we also observed induction of *PsWRKY22* expression in mock samples compared to control (Supplementary Figure S13). *PsWRKY41* expression was strongly inhibited by cutting (Supplementary Figure S13). Our data suggest that *PsWRKY22*, *PsWRKY41,* and *PsPR2* are promising candidate genes for monitoring PTI responses in poppy.

### Callose accumulation in *P. somniferum*

**Figure 4.**
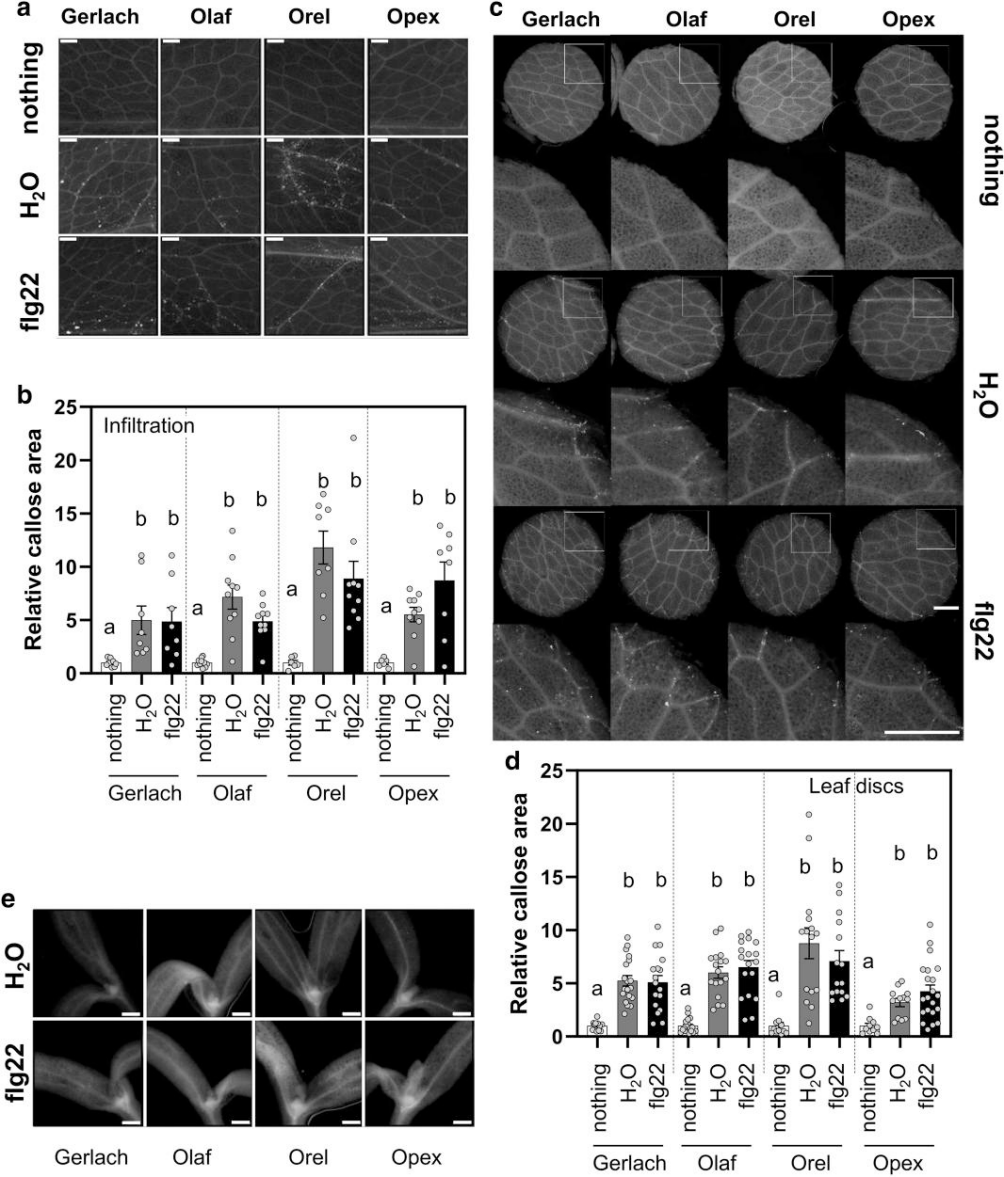
Callose accumulation. (a) Representative images from 5- to 6-week-old poppy leaves treated with 5 µM flg22 (or water) using needleless syringes. (b) Callose accumulation in 5–6 poppy leaves treated with 5 μM flg22 (or water) using needleless syringes (c) Representative images of discs and ROIs from 5- to 6-week-old poppy leaves treated with 5 μM flg22 (or water) using needleless syringes. (d) Callose accumulation in discs cut from 5- to 6-week-old poppy plants. (e) Representative images of 12-day-old poppy seedlings treated with 5 μM flg22 (or water). The callose accumulation was monitored 24 h after treatment. Bars represent 1 mm. The data are presented as mean ± SEM; *n* = 10–16. The experiment was repeated three times (b, e) or two times (d) independently with similar results. Statistical differences between the samples (b, d) were assessed using a one-way ANOVA, with a Tukey honestly significant difference (HSD) multiple mean comparison *post hoc* test. Different letters indicate a significant difference, Tukey HSD, *P* < .01.

Compared to ROS burst and MAPK activation, transient processes which are observable within few minutes after PAMP or DAMP recognition, callose accumulation is generally detected within hours during PTI response (Kalachova et al. 2020, Mason et al. 2020, Liu et al. 2023). We used two approaches for flg22 treatment: (i) infiltration and (ii) leaf disc method. We analysed callose deposition 24 hours after treatment and observed similar callose accumulation levels in response to wounding (water infiltration in infiltrated samples, edge of the leaf discs) and flg22 (Fig. 4a–d). Callose accumulation following flg22 treatment in Arabidopsis is detectable and visible also in seedlings cultivated *in vitro* conditions (Gómez-Gómez et al. 1999, Zahid et al. 2017). This approach overcomes troubles with wounding. Thus, we used poppy seedlings cultivated in *in vitro* conditions and treated them with flg22. However, we did not observe any significant callose accumulation in poppy seedlings cultivated *in vitro* (Fig. 4e). These data indicate that wounding, not flg22, caused increased callose deposition.

### Seedling growth inhibition and reactive oxygen species burst in roots of *P. somniferum*

**Figure 5.**
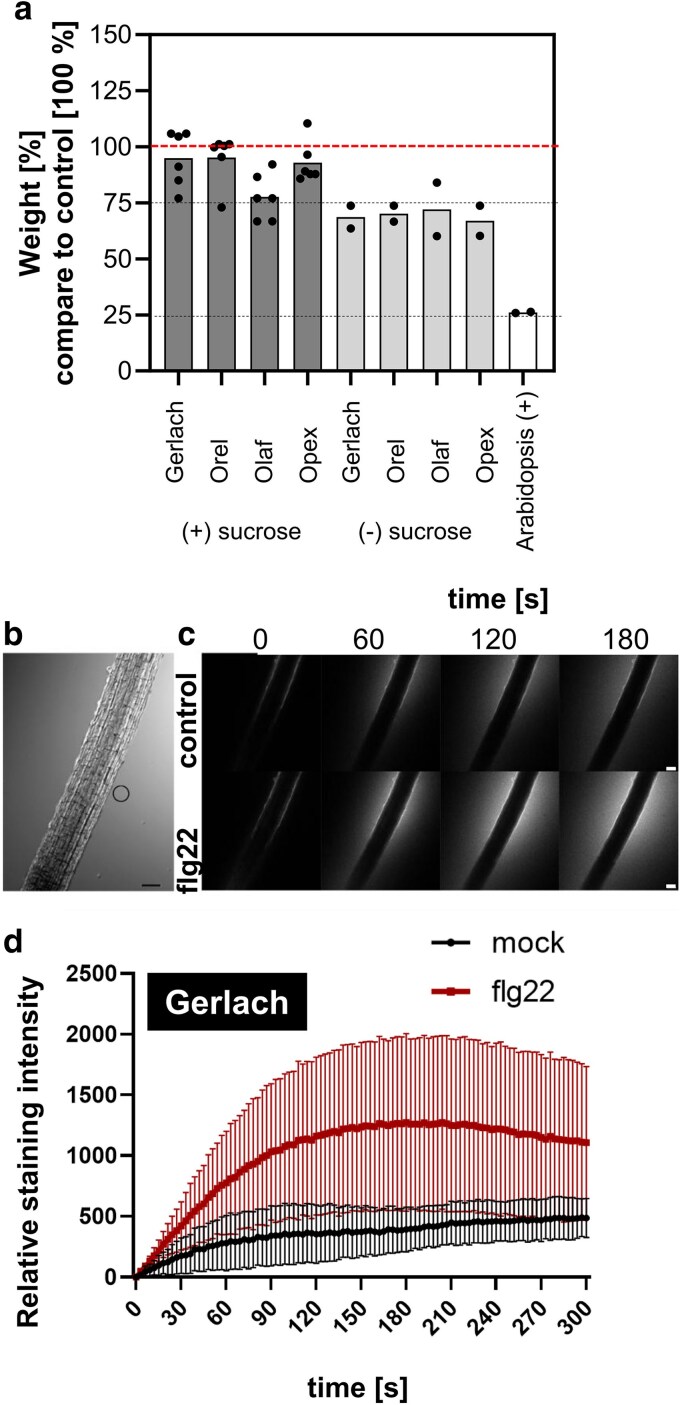
Seedling growth inhibition and ROS production in roots. (a) Growth inhibition analyses. [%] of 5 μM flg22 treated seedlings weight compared to water (control) treated seedlings 5–7 days after the treatment in 1/2 MS medium with vitamins ±1% (w/v) sucrose. The value of control was set as 100% (represented by a red dashed line). Individual values represent the mean from one biological experiment in which was the weight of 10–30 independent seedlings. The bar represents the mean of the means from independent biological experiments. The statistics for each individual independent biological experiment are provided in Supplementary Figures S14 and S15. (b–d) Five-day-old seedlings of Gerlach cultivar were treated with 1 μM flg22. (b) Representative image (in the bright field) of the poppy root, outlined area denotes the ROI, from which measurements were taken. (c) Representative images of Amplex Red signal (the halo around the root) caused by extracellular ROS burst after 1 μM flg22 treatment. Scalebar =100 μm. (d) Analysis of Amplex Red and ROS accumulation induced fluorescence in the root elongation zone upon treatment with 1 μm flg22 *n* ≥ 4 (independent samples within one biological repeat), Data are presented as mean ± SD. (d) was done in total in three biological repeats with similar results.

A typical long-term effect of plant immunity is the inhibition of plant growth, resulting from the so-called growth-defence trade-off (He et al. 2022). The screening is usually done with seedlings grown in liquid media *in vitro* (Gómez-Gómez et al. 1999, Janda et al. 2023). We used the same approach with poppy using liquid media containing sucrose (see Material and methods). Unlike Arabidopsis, flg22 did not significantly inhibit the growth of poppy seedlings (Fig. 5a and Supplementary Figure S14). Only the Olaf cultivar showed statistically significant growth inhibition, around 20%, in four out of six independent experiments (Fig. 5a and Supplementary Figure S14). However, we also tested the growth inhibition in sucrose-free liquid media, and in such an experimental design we observed significant growth inhibition in all cultivars in all independent experiments (Fig. 5a and Supplementary Figure S15). The inhibition was around 25%, significantly weaker than Arabidopsis (Fig. 5a).

Observing no callose accumulation (Fig. 4e), no ROS production (Supplementary Figure S16), and weaker (growth inhibition) responses in *in vitro* experimental conditions, we searched for another possible screening method for PTI analysis in seedlings. For this purpose, we used the method developed by Kulich et al. (2025) to analyse apoplastic ROS burst in roots using Amplex Red dye. The reaction provides red fluorescence upon oxidation to resorufin in the presence of H_2_O_2_. The advantage of the method is that it monitors very fast response and allows each sample to serve as its own control. We observed clear apoplastic ROS production (visible as a halo around the poppy roots; Fig. 5b and c) within 5 minutes after treatment with flg22 (Fig. 5d).

### Ethylene and salicylic acid production in *P. somniferum*

**Figure 6.**
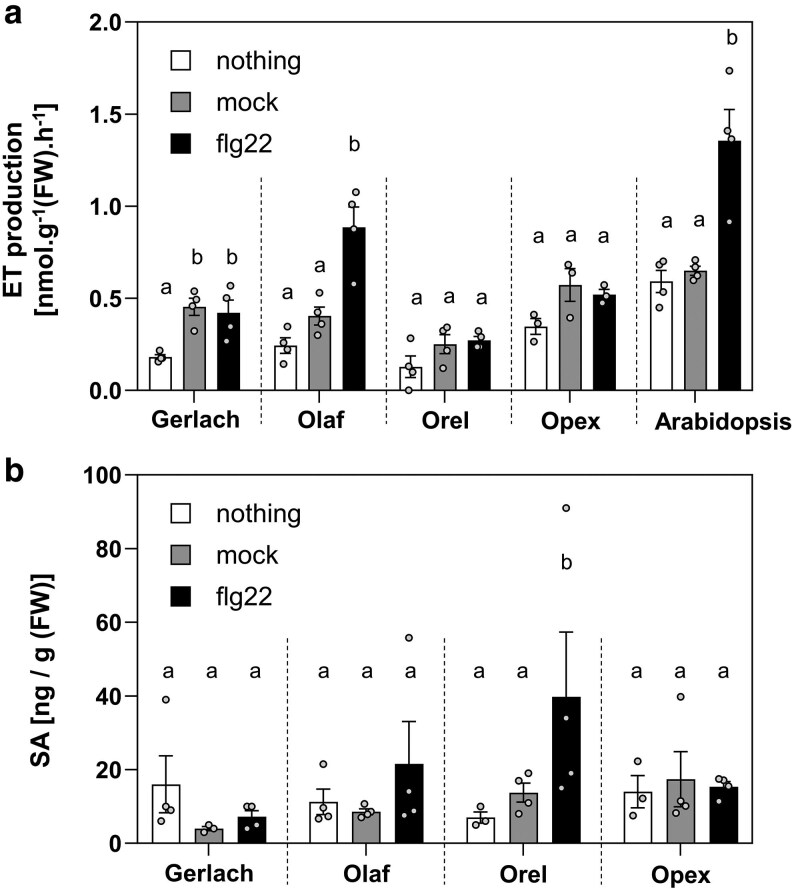
Ethylene and salicylic acid accumulation. The leaves from 5- to 6-week-old poppy and *A. thaliana* plants were treated with 5 μM flg22 with infiltration using a needleless syringe. As controls, untreated leaves and leaves treated with water (mock) were used. (a) The ethylene production from poppy and Arabidopsis leaves was analysed 4 h after treatment. (b) Salicylic acid (SA) concentration was analysed 24 h after the treatment with flg22. The data are presented as mean ± SEM; *n* = 3–4 (ethylene) and *n* = 4 (SA). The ethylene experiment was repeated three times with similar results. Statistical differences between the samples among one genotype were assessed using a one-way ANOVA, with a Tukey honestly significant difference (HSD) multiple mean comparisons *post hoc* test. Different letters indicate a significant difference, Tukey HSD, *P* < 0.01.

Production of ethylene, a gaseous defence-related phytohormone, is a fast and rapid response to flg22 recognition in Arabidopsis (Felix et al. 1999). Using a needleless syringe, we treated poppy leaves with 5 µM flg22 and distilled water as mock control. We repeatedly observed significantly increased ethylene production as a response to wounding, which was caused by the infiltration of the water in the Gerlach cultivar (Fig. 6a). In Opex and Orel, the trend was similar, but the difference was not significant. In the Olaf cultivar, we observed a significant difference between water and flg22 treatment (Fig. 6a).

Additionally, we analysed another typical defence-related phytohormone, salicylic acid (SA). For this purpose, we used UPLC with a fluorescence detector inspired by a method published in a study measuring SA in poppy after endophyte inoculation (Ray et al. 2021). However, we were unable to detect any SA. Thus, we used the method we previously, successfully, used for Arabidopsis analyses (Pluhařová et al. 2019). With this method, we identified SA in the range of 5–40 ng/g of fresh weight tissue (Fig. 6b). However, we did not observe any effect caused by wounding. Only for Orel cultivar did the treatment with flg22 show a significant increase in SA concentration (Fig. 6b). The SA content in poppy was low compared to our Arabidopsis data (around 1–2 μg/g FW) (Hao et al. 2012, Pluhařová et al. 2019) or levels observed in other plant species (0.3–1 μg/g FW in *Humulus lupulus*, 1–3 mg/g FW in willow bark) (Petrek et al. 2007, Patzak et al. 2013).

## Discussion

Research on *Papaver somniferum* L. (poppy) has been so far predominantly focused on biosynthetic pathways and the production of secondary metabolites (Singh et al. 2019). It was shown that poppy, particularly *Papaver rhoeas*, is suitable as a model for studying cell death, especially in pollen (Garcia-Alonso et al. 2022, Geitmann et al. 2004, Wilkins et al. 2015). Importantly, only few studies were focused on the molecular aspects of the interactions between poppy and its pathogens (Thangavel et al. 2020, Ray et al. 2021) although poppy yield is strongly influenced by pathogens and pests (Bailey et al. 2000, Thangavel et al. 2018). To the best of our knowledge, no analysis has been focused on plant immunity in poppy, especially on a molecular level. Here, we provide the first comprehensive investigation of PTI in poppies.

As a base cultivar for analysing breadseed poppy PTI responses, we selected the spring blue seed cultivar Gerlach, which was introduced into the market in 1990. Using Gerlach, we showed that, among all tested peptide elicitors, flg22 is the most potent peptide to induce ROS burst (Fig. 1). However, flg22-triggered ROS production was significantly lower in Gerlach compared to Arabidopsis (Fig. 1e). This could be explained by a lower binding affinity of *Ps*FLS2 to flg22 compared to the binding affinity of *At*FLS2, which we predicted in modelled bioinformatic analysis (Fig. 2a). Another explanation could be the composition of the leaves, such as cuticle composition; for example, *A. thaliana* has a total wax coverage of ∼0.7–1.5 μg cm^−2^ (Lee and Suh 2015), whereas *P. somniferum* leaves have 194 μg cm^−2^ (Jetter and Riederer 1996). Our preliminary measurements of total wax composition (measurement were performed using method published in Kubásek et al. 2023) confirm the published data for Arabidopsis (∼0.5 μg cm^−2^), however for poppy (cultivar Gerlach) leaves the total wax composition was ∼20 μg cm^−2^ which is lower than was shown by Jetter and Riederer (1996), but is still 40 times higher than for Arabidopsis. Thus, flg22 might have reduced accessibility to poppy leaves compared with Arabidopsis. We showed that in poppy flg22 induced putative MAPK phosphorylation (Fig. 3b and c), seedling growth inhibition (Fig. 5a), ROS burst in roots (Fig. 5b) and we identified candidate genes for monitoring PTI (Fig. 3d). Surprisingly, compared to other plant species (Tsuda et al. 2008, Nguyen et al. 2010, Skottke et al. 2011, Lloyd et al. 2014), no callose accumulation (Fig. 4), ethylene (Fig. 6a) or SA (Fig. 6b) production was observed after flg22 in Gerlach. This might also be because of the leaf composition, but mechanisms such as a distinct regulation of callose accumulation cannot be excluded as a possibility. However, we have not analysed, within this study, the reasoning behind this behaviour in detail.

We compared the PTI responses of Gerlach with other poppy cultivars which were selected to represent the common breadseed poppies on the fields in Central Europe: spring white seed (Orel), overwintering blue seed (Olaf), and another spring blue seed (Opex). Most of the PTI responses were similar in all cultivars compared to Gerlach, with few exceptions. Gerlach had stronger ROS responses to flg22 in three out of five biological repeats in comparison with Opex and Olaf cultivars; in two out of five repeats response in Gerlach was stronger compare to Orel (Supplementary Figure S3). In contrast to Gerlach, we observed increased SA concentration in Orel in response to flg22 (Fig. 6b). Interestingly, SA concentration in poppy cultivars is at least ten times lower than in other plant species (Hao et al. 2012). Thus, deciphering the potential role of SA in poppy defence against pathogens should be further investigated. For example, SA signalling can be monitored with transcriptomic analysis or testing of the SA treatment on poppy growth and defence against pathogens. Unlike other cultivars, Olaf was the only cultivar in which flg22 triggered a significant increase in ethylene production (Fig. 6a). Olaf also exhibits growth inhibition after flg22 treatment in media containing sucrose (Fig. 5a) and has a slower induction of ROS production (Supplementary Figure S5). Spring cultivars might have different strategies for defence compared to overwintering cultivars. Having more spring and overwintering blue-seeded cultivars in a germplasm seed bank opens research opportunities which might be unique among other plant species.

Analysis of MAPK phosphorylation and callose accumulation revealed that wounding stress is a challenge in PTI investigation in poppy. Whereas, in comparison to studies performed on *Brassicaceae* or *Solanaceae*, in which syringe infiltration was successfully used (Nguyen et al. 2010, Lloyd et al. 2014). In poppy, syringe infiltration resulted in a similar level of putative MAPK phosphorylation for water-treated samples as for flg22-treated ones (Fig. 3a). Using a leaf disc approach, we overcame this problem (Fig. 3b). Monitoring callose, we observed significant callose accumulation after syringe infiltration of water at the same level as for flg22-treated samples in the site of infiltration (Fig. 4). Additionally, using the leaf disc approach we observed callose accumulation near the cut region, but no differences between water- and flg22-treated samples (Fig. 4). It remains unclear whether the lack of difference between water and flg22-treatment was because in poppy callose accumulation is not part of the PTI repertoire or if we did not succeed in identifying optimal conditions for studying callose accumulation in response to flg22 treatment in poppy. A proposal to alternative protocols can be found here (Tee et al. 2023).

To overcome the problem with wounding, we used seedlings grown *in vitro* for analysis (Supplementary Figure S2), but we did not observe callose accumulation in seedlings (Fig. 4) even though we did not observe ROS burst in seedlings using the luminol-based method (Supplementary Figure S16). Nevertheless, we used a recently developed assay for monitoring extracellular ROS burst in seedlings roots using Amplex Red fluorescent dye (Kulich et al. 2025). With this approach, we observed clear induction of ROS burst caused by flg22 treatment in roots (Fig. 5b and c). This method seems to have great potential for screening the root reaction to molecular patterns, and it would be great to incorporate it among the set of methods for PTI studies, especially in poppy. Additionally, in seedlings, we observed growth inhibition after flg22 treatment. To note the reproducible inhibitory effect of flg22, we observed just using a medium without sucrose (Fig. 5a). This is the methodologically relevant observation that such a compound as sucrose, which is commonly used in the Arabidopsis research (Wierzba and Tax 2016, Janda et al. 2023), can abolish the effects of flg22 in poppy.

The response of poppy to wounding represents the opportunity for deeper analysis PTI. PTI is interconnected with wounding, sharing similar responses on the molecular level (Choi and Klessig 2016). PAMPs represent bacterial molecules inducing immunity, but during infection, plants produce DAMPs, molecules whose production is triggered by damage of plant tissue (Hou et al. 2019). Our study used one DAMP: *At*Pep1; a peptide originating from Arabidopsis (Huffaker et al. 2006). Based on the available literature, *At*Pep1 might be specific for Arabidopsis (*Brassicaceae*). However, we monitored the weak induction of ROS burst after *At*Pep1 treatment in poppy leaves (Fig. 1, S3). Investigating the presence of DAMP peptides similar to *At*Pep1 in poppy will be interesting.

We showed that with established methods, it would be possible to perform systematic screenings of poppy PTI responses to other known elicitors, such as chitin, to which the immune response is even more conserved in plants than to flg22 (Gimenez-Ibanez et al. 2019). Importantly, the presented methodology can be used for the identification of novel elicitors triggering poppy immunity. The obtained knowledge would streamline future poppy breeding for better resistance to pathogens. For example, the information that poppy does not respond to known PAMPs or DAMPs with a known receptor in other plant species could potentially enable us to design a way to obtain a novel transgenic poppy resistant to a particular pathogen using an approach similar to the introduction of EFR into plant species from families other than *Brassicaceae* (Lacombe et al. 2010, Schwessinger et al. 2015, Schoonbeek et al. 2015, Lu et al. 2015, Boschi et al. 2017, Mitre et al. 2021, Piazza et al. 2021, Adero et al. 2023). Additionally, screening PTI responses across available poppy cultivars (e.g. from Czech poppy seeds bank (https://grinczech.2025.cz) or performing EMS mutagenesis screens could further enable us to decipher PTI signalling in poppy.

## Conclusion

Our study provides a methodological pipeline for studying PTI and illustrates its complexity in poppy. Activated PTI after flg22 treatment is evident, as suggested by ROS burst, putative MAPK phosphorylation, seedling growth inhibition, and altered gene transcription. Additionally, ethylene and SA production also increased in certain cultivars. Interestingly, callose accumulation seems to be independent of flg22 treatment. Our results indicate that attention must be paid to overlaps between PTI and wounding when investigating PTI. The insights and methodological know-how gained from this research not only advance our understanding of plant immunity in poppy, but also open new possibilities for future studies that aim to improve disease resistance in poppy. Further research should explore the genetic basis of the observed variability and investigate the role of additional signalling molecules to develop comprehensive strategies for enhancing the resilience of poppy against various pathogens.

## Supplementary Material

plaf055_Supplementary_Data

## Data Availability

The authors confirm that the data supporting this study's findings are available within the article and its supplementary materials. The raw data files for all experiments are available upon request from the corresponding author.
