# Supplementary material for: Pattern-triggered immunity in blue and white seed cultivars of Papaver somniferum
Source: AoB Plants. 2025 Oct 6;17(5):plaf055. doi: 10.1093/aobpla/plaf055 (PMC12560825; doi:10.1093/aobpla/plaf055)
Supplement: plaf055_Supplementary_Data [file plaf055_supplementary_data.pdf]

SUPPLEMENTARY MATERIAL

Hernández Orozco et al. 2025

Pattern-triggered immunity in blue and white seed cultivars of *Papaver somniferum*

Figure S1. Representative photos of *Papaver somniferum* plants growth in soil from 5th to 30th day.

Figure S2. Representative figures of *Papaver somniferum* growth in sterile conditions from seed germination to 10th day.

Figure S3. ROS burst in poppy after treatment with elicitors.

Figure S4. ROS burst in poppy cultivars triggered by treatment with flg22.

Figure S5. Positive control for triggering ROS burst by the peptides in distinct potatoes and Arabidopsis.

Figure S6. ROS burst in Gerlach and Arabidopsis triggered by treatment with distinct concentrations of flg22.

Figure S7. Alignment of *At*FLS2 (AT5G46330.1) and *Ps*FLS2(XP_026454075.1) protein sequences.

Figure S8. Confidence plots of 3D *Ps*FLS2 predicted structure.

Figure S9. ROS burst in Gerlach triggered by treatment with distinct flg22.

Figure S10. Response to flg22 originating from *Xanthomonas campestris* pv. *Campestris* – data from rest biological repeats

Figure S11. Original documentation of full gels of immunoblots which were shown in figure 3.

Figure S12. Gene expression after treatment with flg22.

Figure S13. Gene expression after treatment with H_2O compared to non-treated samples.

Figure S14. Raw data for seedlings growth inhibition in media containing sucrose shown in figure 5.

Figure S15. Raw data for seedlings growth inhibition in media without sucrose shown in figure 5.

Figure S16 ROS burst in seedlings of four poppy cultivars after flg22 treatment.

Table S1. Primers used for RT-qPCR in *P. somniferum*

Table 2. List of binding affinity energies for *P. somniferum* and *A. thaliana* FLS2 and flg22 and *Xcc*Flg22

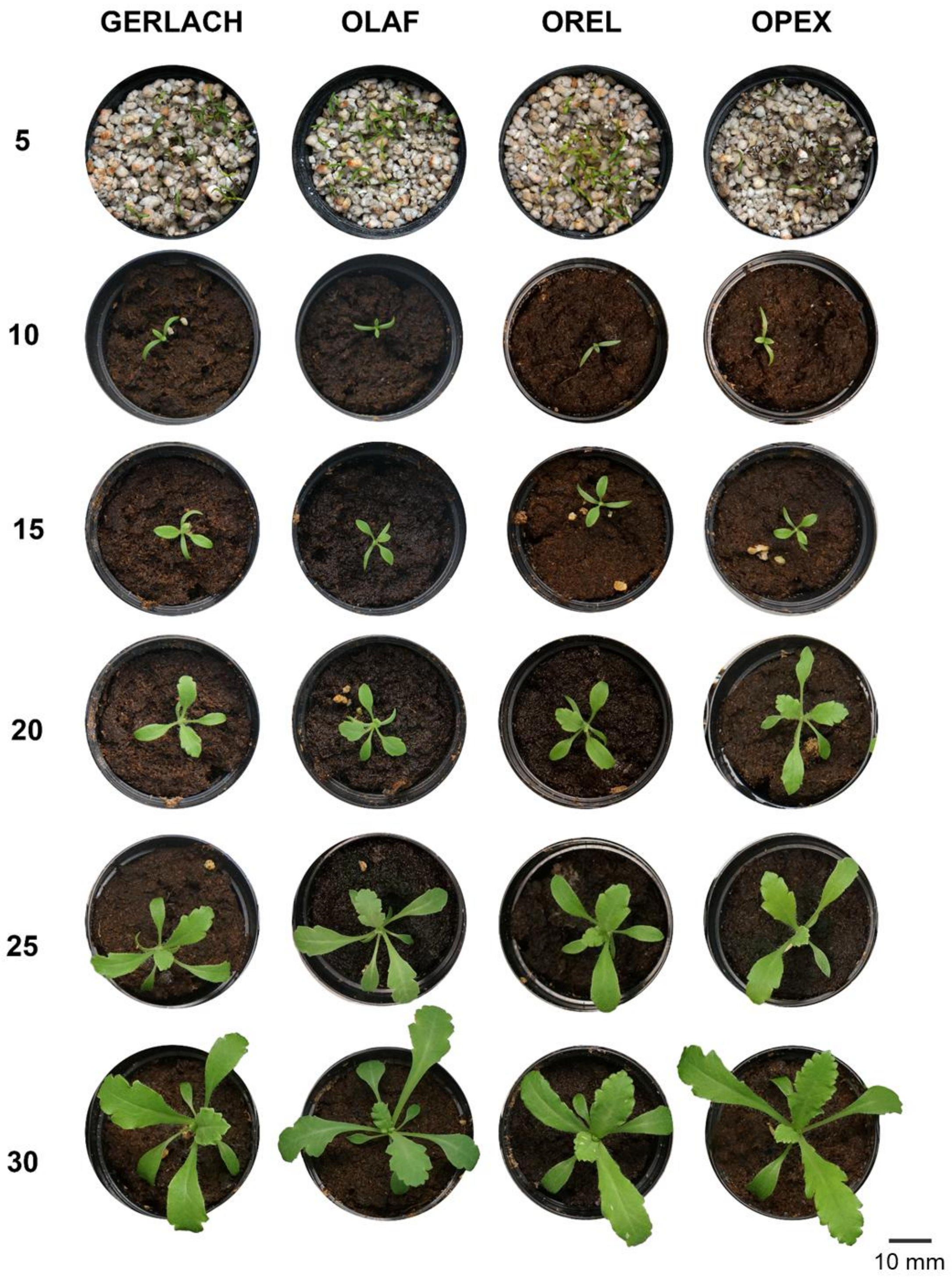

Figure S1. Representative photos of *Papaver somniferum* plants growth in soil from 5th to 30th day. (until 5th day plants were grown in perlit in hydroponia)

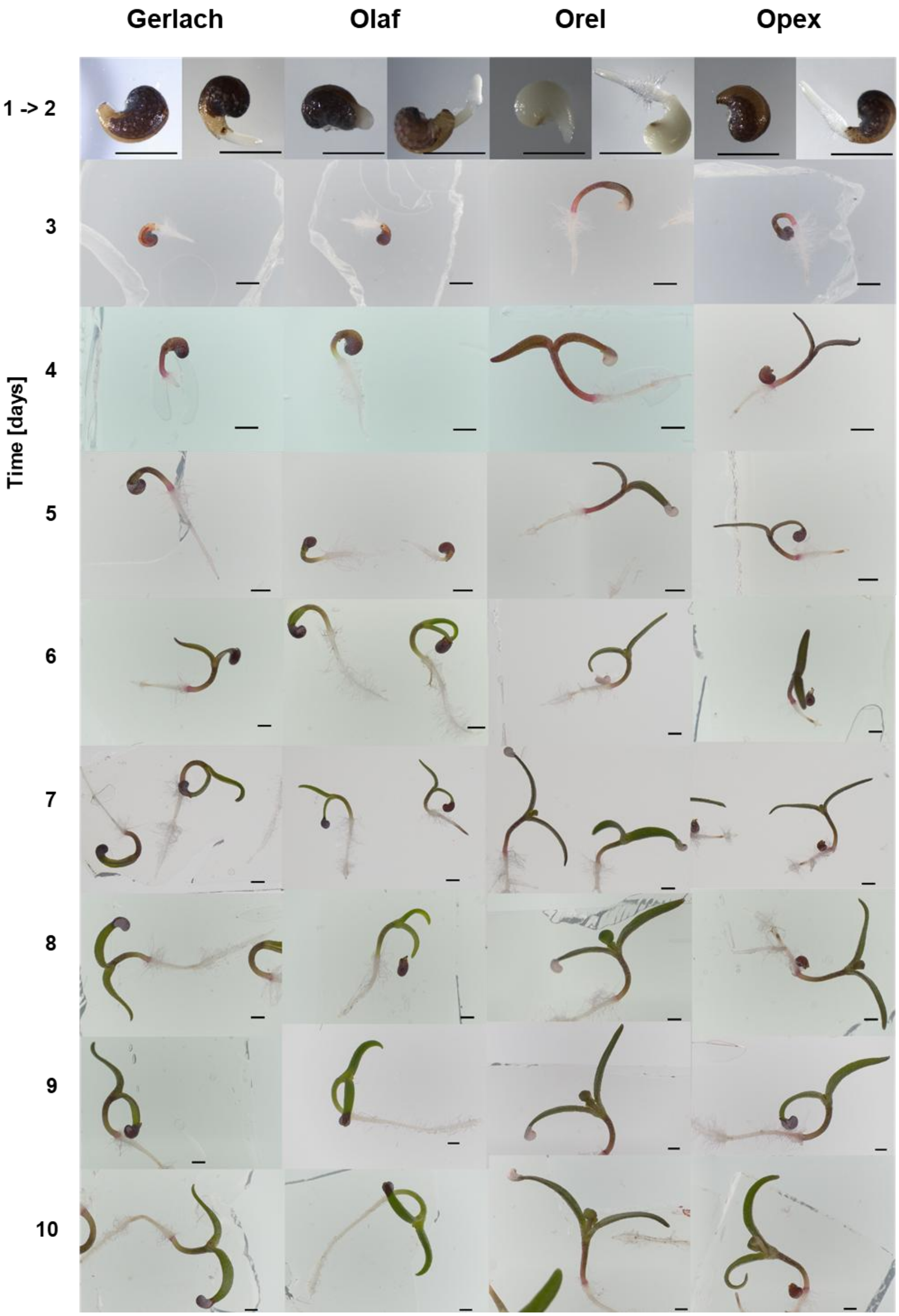

Figure S2. Representative figures of *Papaver somniferum* growth in sterile conditions from seed germination to 10th day. The bar represents 1 mm.

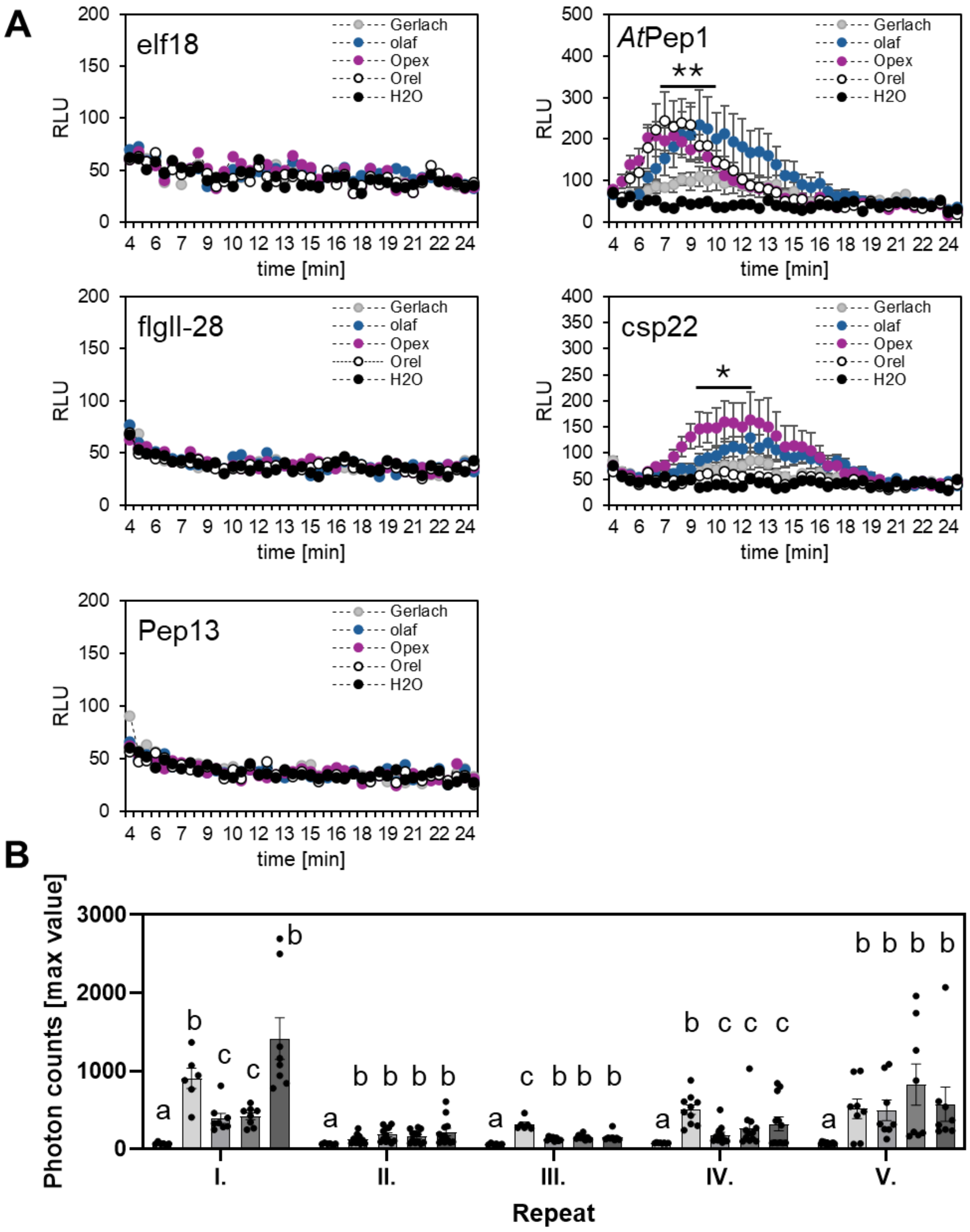

Figure S3. ROS burst in poppy after treatment with elicitors. Discs were cut from 5-6 week old poppy plants. **A)** Discs were treated with 1 µM: flgII-28, csp22, *At*Pep1, elf18, Pep13. The experiment was repeated three times independently with similar results. **B)** Data from five biological repeats for all cultivars treated with 1 µM flg22. The data represent the means; n=8-16 discs in one biological experiment (A, B). Asterisks (A) indicate that the mean value is siginificantly different from the control (H_2O) conditions (two-tailed Student´s t-test *$p<0.05$, **$p<0.01$). The data represent the means; n=8-16 discs in one biological experiment. Statistical differences between the samples (B) were assessed using a one-way ANOVA, with a Tukey honestly significant difference (HSD) multiple mean comparison post hoc test. Diferent letters indicate a signifcant diference, Tukey HSD, $p<0.05$.

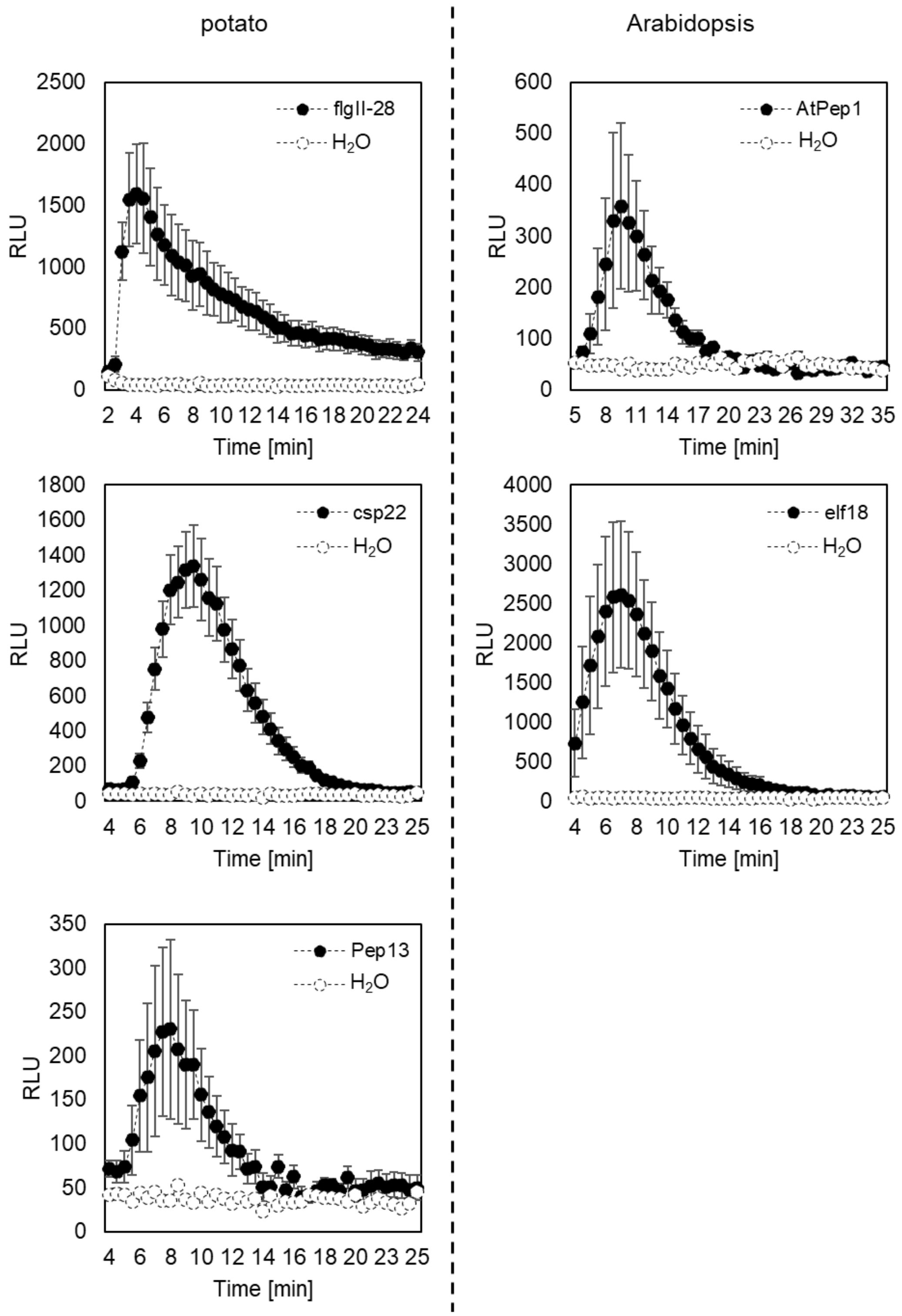

Figure S4. Positive control for triggering ROS burst by the peptides in distinct potatoes and Arabidopsis. Discs were cut from potato (Pep13, csp22, FlgII-28), Arabidopsis (elf18, *At*Pep1). The data represent mean and SEM from 8-12 discs. The experiment was repeated two times with similar results.

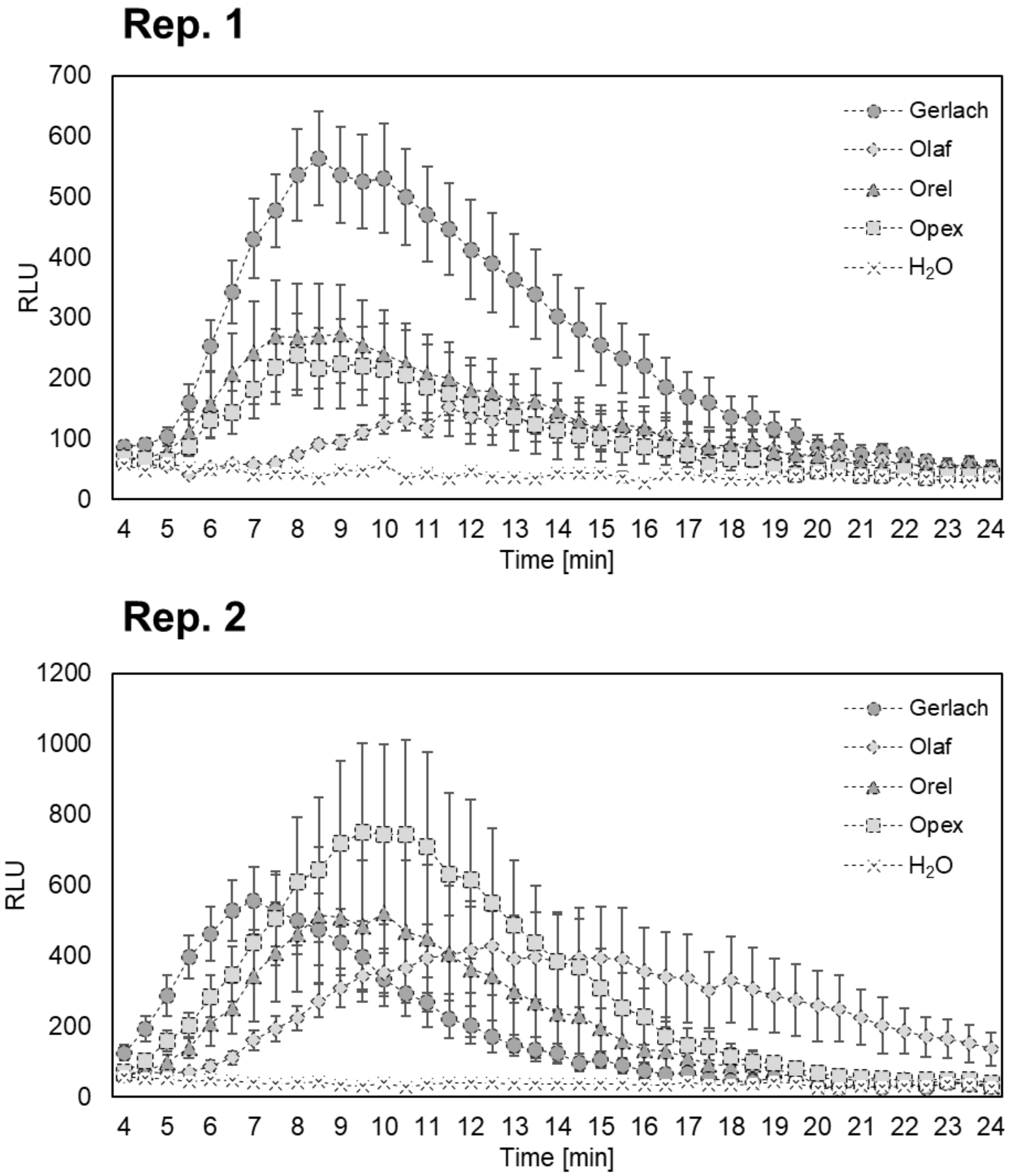

Figure S5. ROS burst in poppy cultivars triggered by treatment with flg22. Discs were cut from 5-6 week old poppy. The data represent the means and SEM; n=8-16 discs. Two biological experiments are shown.

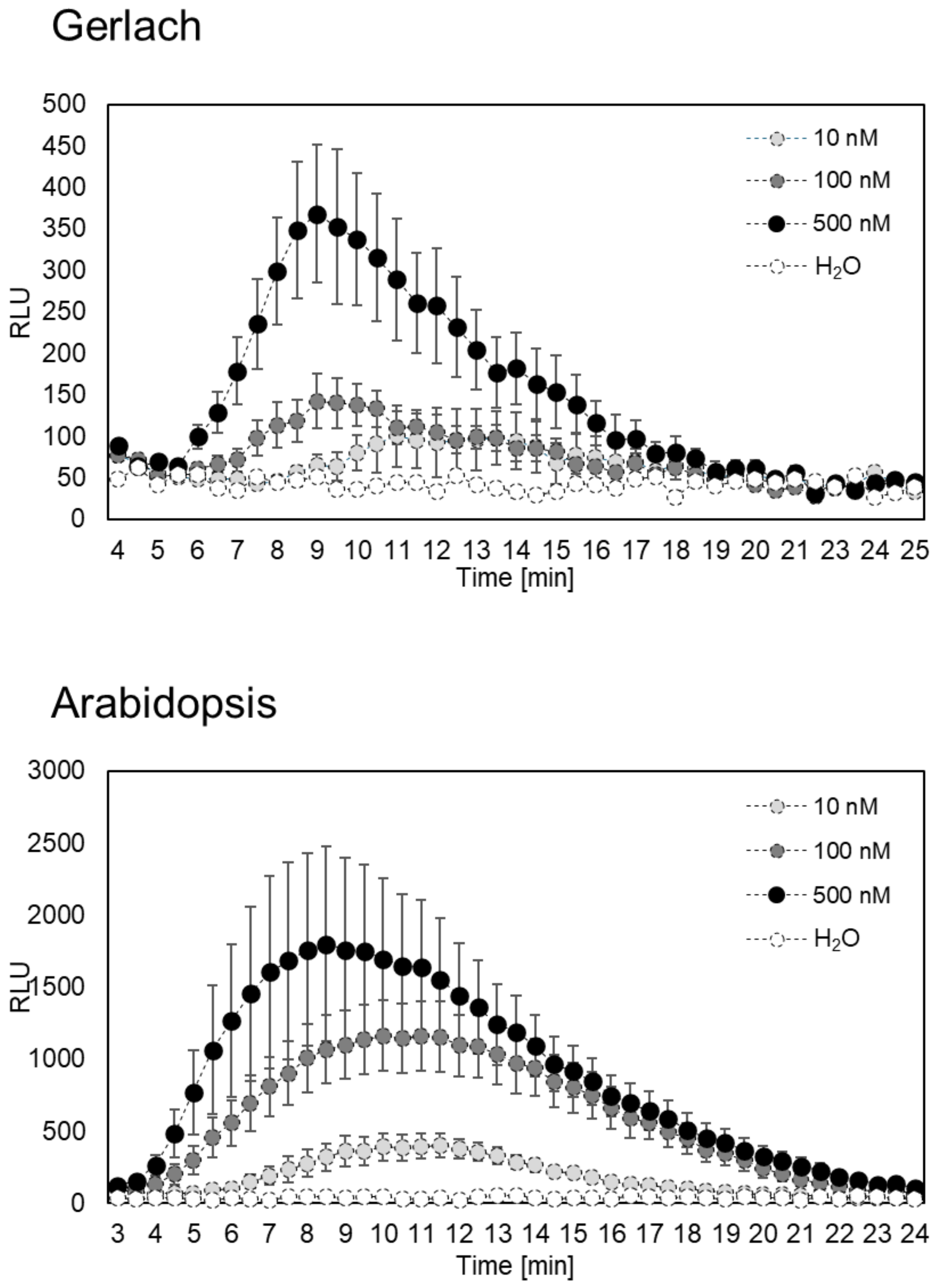

Figure S6. ROS burst in Gerlach and Arabidopsis triggered by treatment with distinct concentrations of flg22. Discs were cut from 5-6 week old Gerlach and *A. thaliana.* The data represent the means and SEM; n=8-16 discs. The experiment was repeated in two biological experiments with similar results.

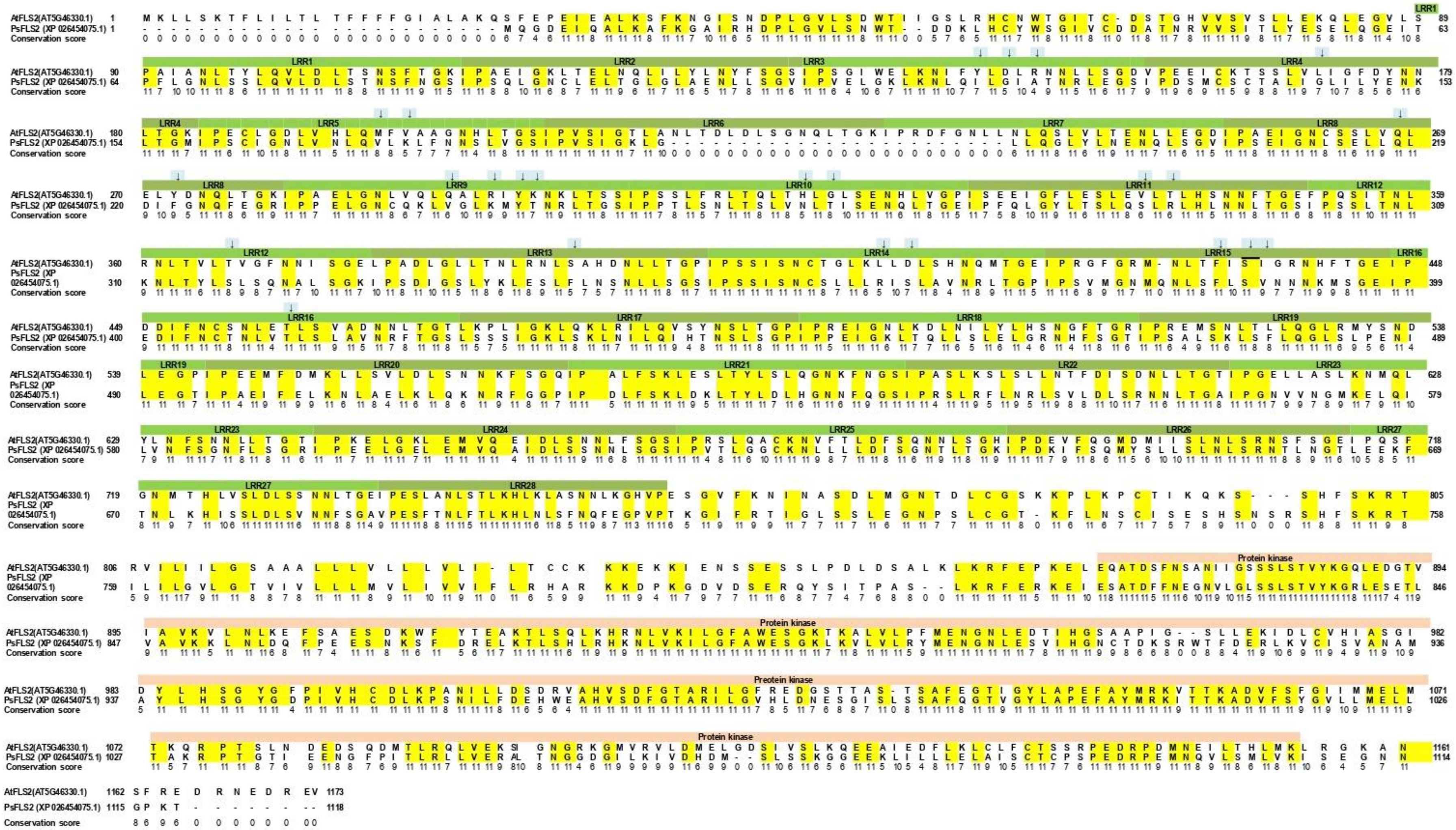

Figure S7. Alignment of *At*FLS2 (AT5G46330.1) and *Ps*FLS2(XP_026454075.1) protein sequences. Yellow color represent the conserved amino acid regions, LRRs are represented with green, light orange represents the protein kinase domain, and potential binding amino acids with flg22 are pointed with an arrow on light blue background.

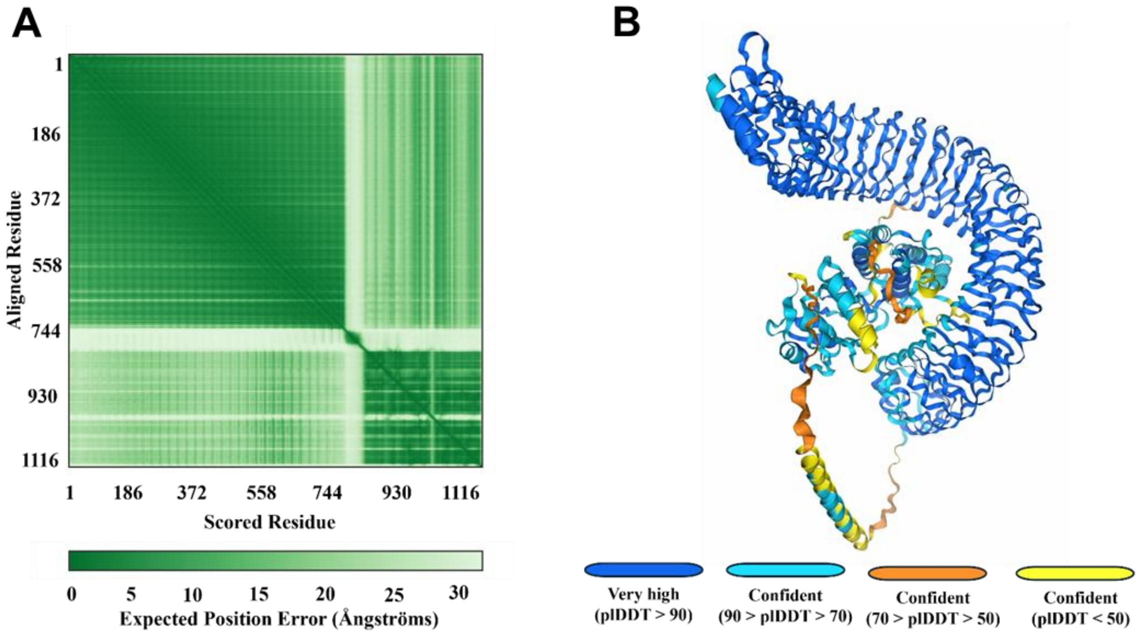

Figure S8. Confidence plots of 3D *Ps*FLS2 predicted structure. A) Predicted Aligned Error (PAE) plot for predicted *Ps*FLS2. **B)** Predicted local Distance Difference Test **(**pLDDT). Both plots were generated automatically on AlphaFold2 server version 2.3.0.

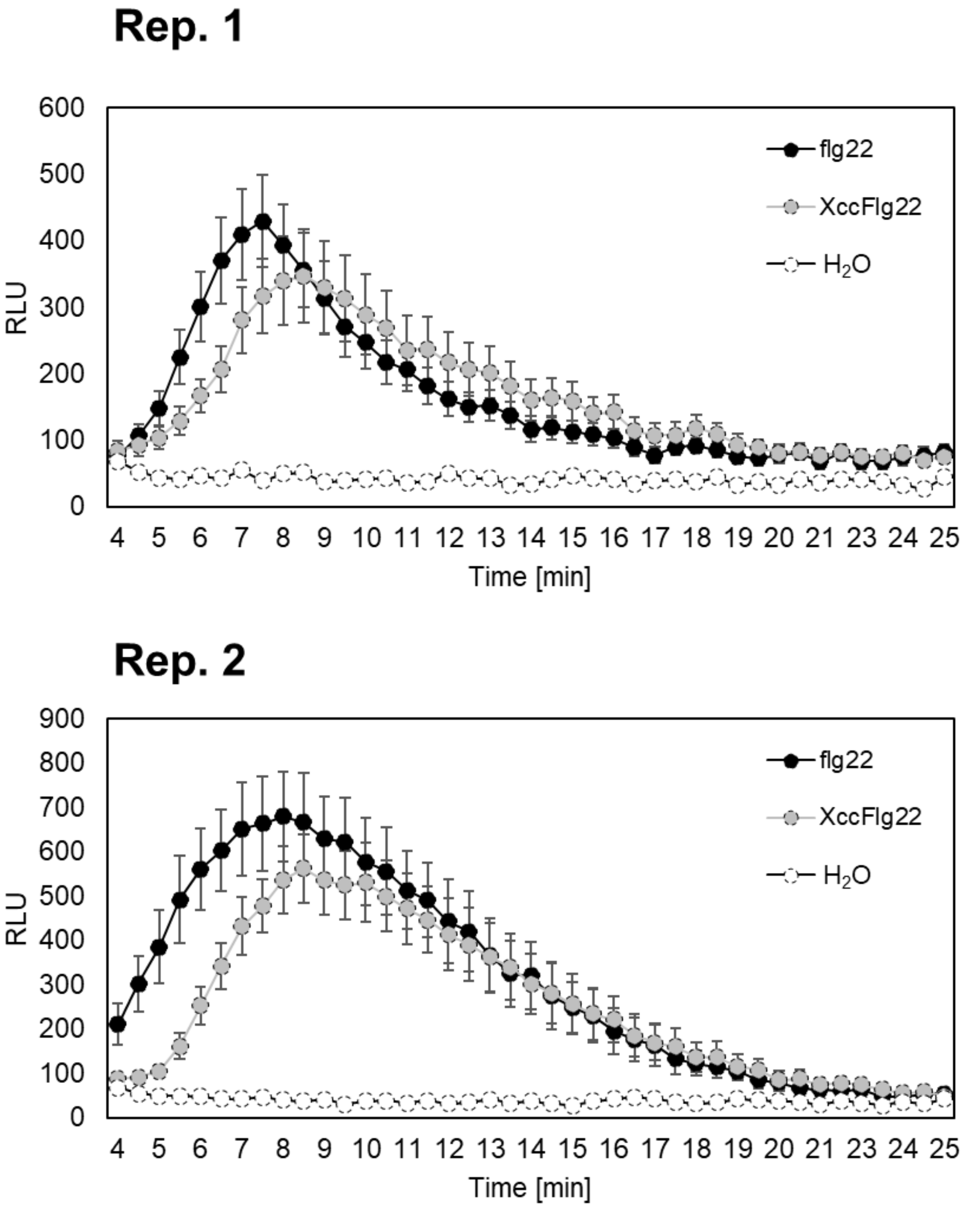

Figure S9. ROS burst in Gerlach triggered by treatment with distinct flg22. Discs were cut from 5-6 week old Gerlach and *A. thaliana.* The data represent the means and SEM; n=8-16 discs. The show two biological experiments.

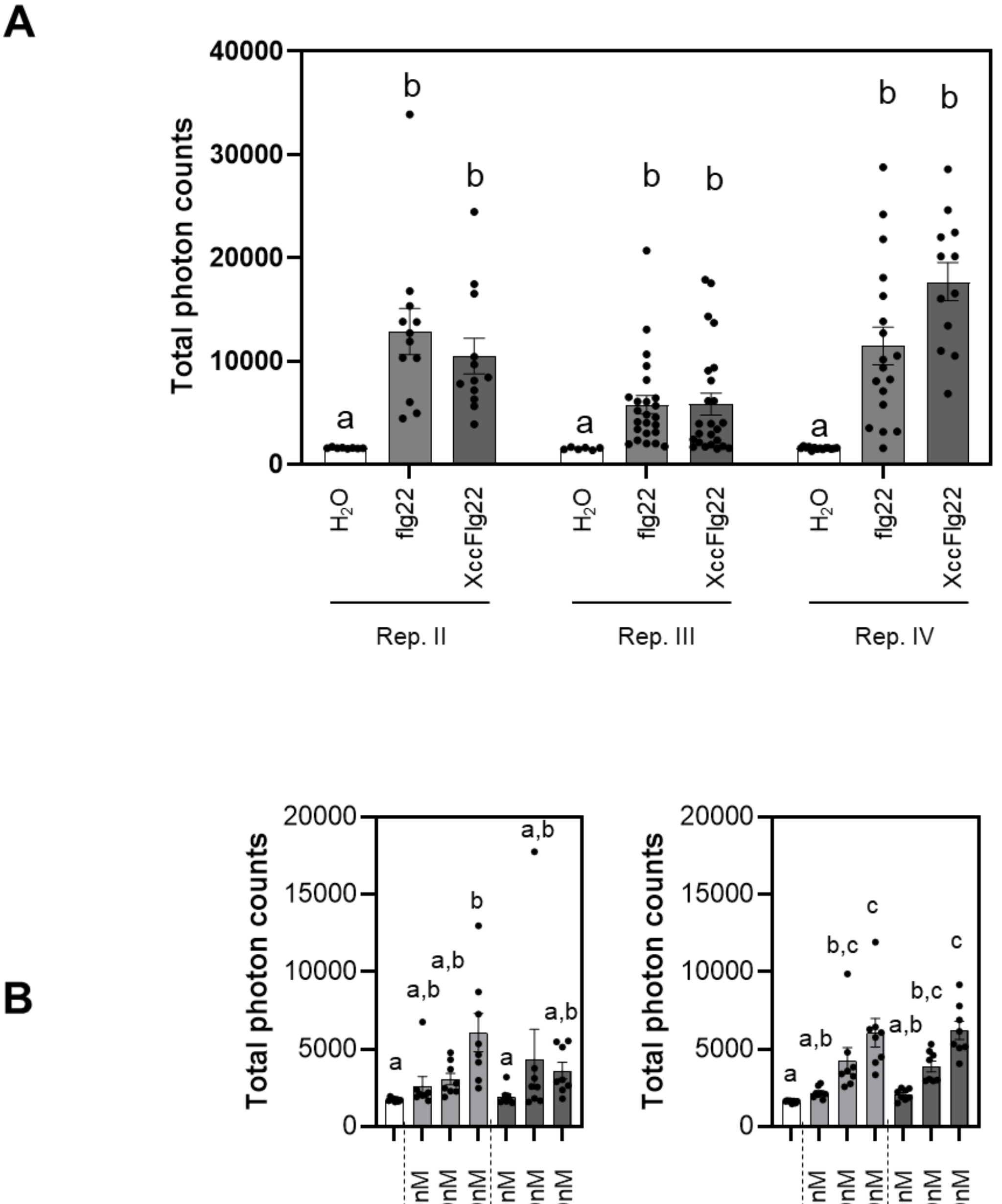

Figure S10. Response to flg22 originating from *Xanthomonas campestris* pv. *Campestris* – data from rest biological repeats (supplement for figure 2). **A-B)** Discs from Gerlach were cut from 5-6 week-old poppy plants. Discs were treated with 1 µM flg22 or XccFlg22 **(A)** and 10 – 500 nM flg22 or XccFlg22 **(B)**. The data represent the means + SEM; n=8-16 discs in one biological experiment. Statistical differences between the samples **(D, E)** were assessed using a one-way ANOVA, with a Tukey honestly significant difference (HSD) multiple mean comparison post hoc test. Different letters indicate a significant difference, Tukey HSD, $P<0.05$. The results from Rep. I for both **(A, B)** are shown in figures 2D and 2E.

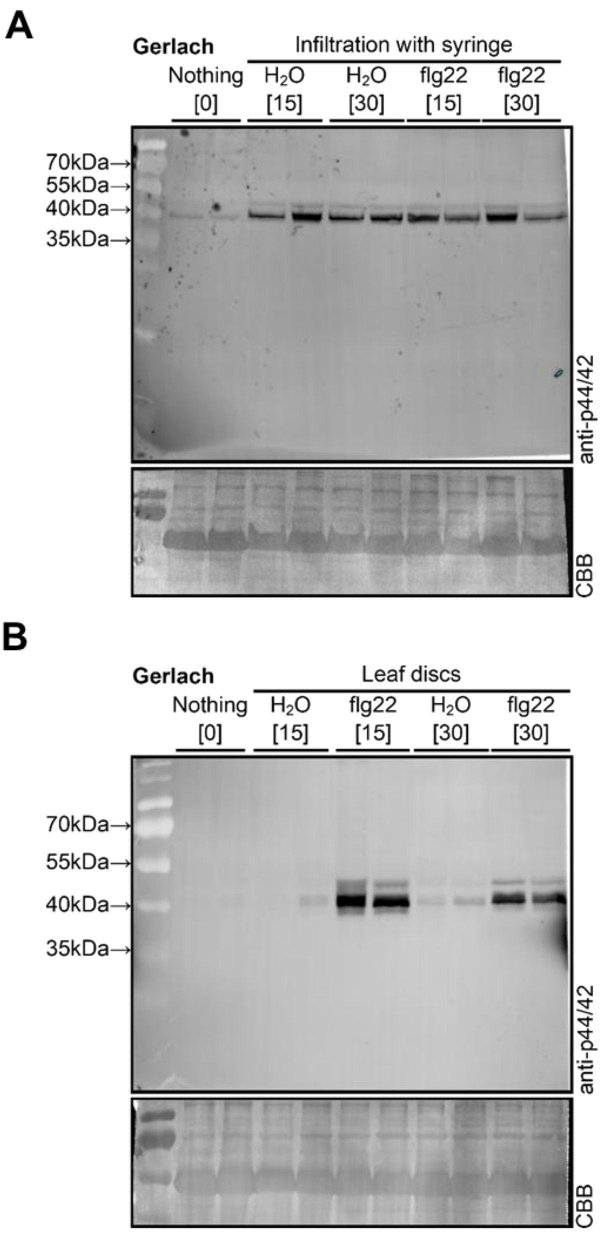

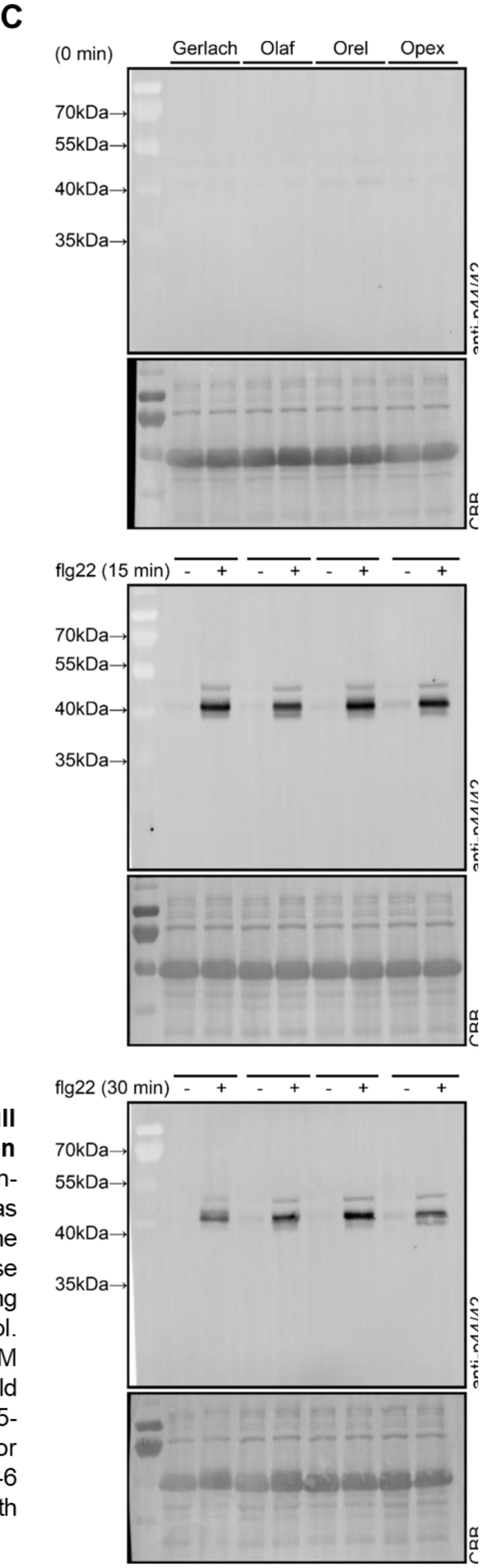

Figure S11. Original documentation of full gels of immunoblots which were shown in figure 3. The activation of the putative mitogen-activated protein kinases (MAPKs) was visualized by western blot analysis using the the pERK phospho-p44/42 MAP kinase antibody. Comassie briliant blue (CBB) staining of the membrane served as a loading control. **A)** Needleless syringae infiltration with 5µM flg22 (or water) of leaves from 5-6 week old poppy plants. **B)** Treatment of leaf discs from 5-6 week old Gerlach plants with 5µM flg22 (or water). **C)** Treatment of leaf discs from 5-6 week old poppy plants (four genotypes) with 5µM flg22 (or water).

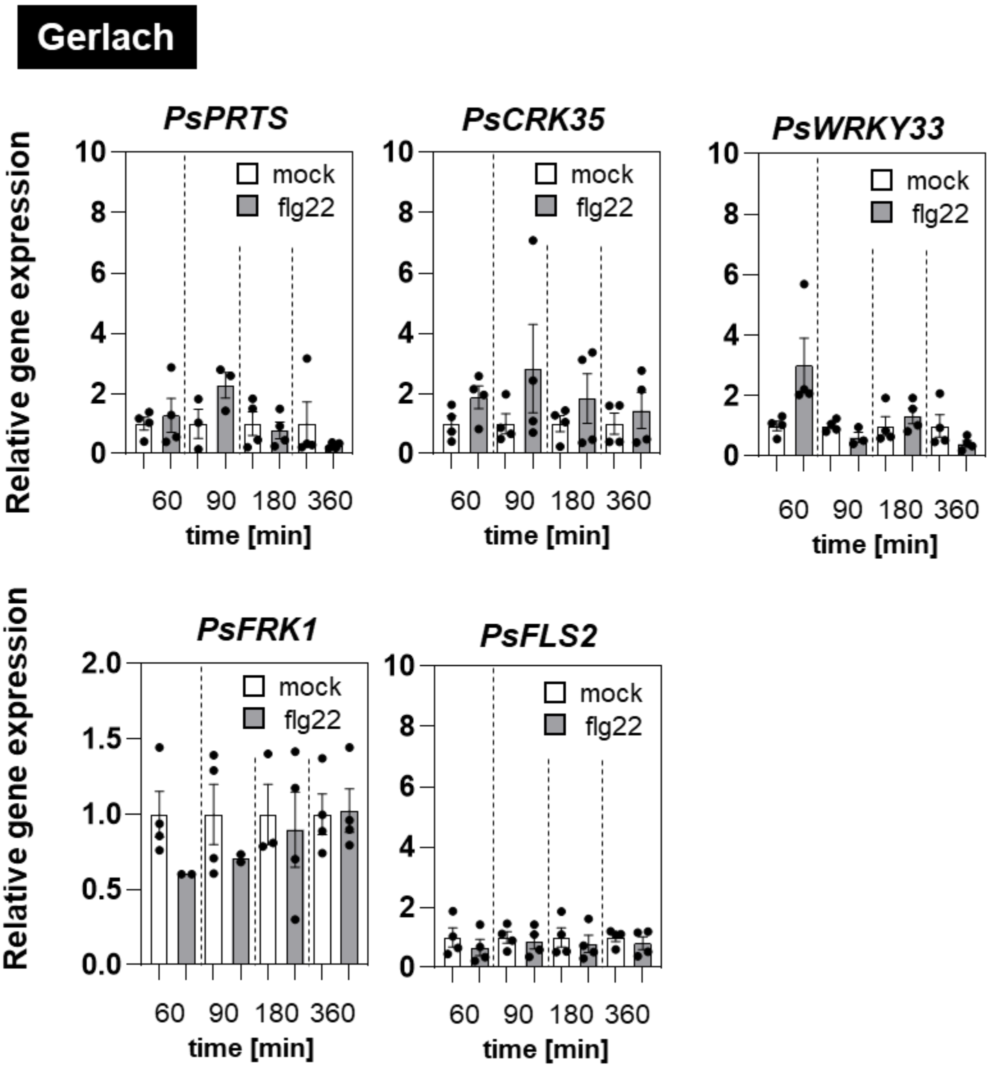

Figure S12. Gene expression after treatment with flg22. The leaf discs were cut from 5-6 week old poppy and treated with 5 µM flg22 for 60, 90, 180, 360 minutes. Expression values were normalized to the mock samples (water treated), which were set to 1. Expression levels in treated samples are presented relative to the corresponding control (mock in the same time). The data represent the means + SEM; n=3-4. Mock and flg22 treated samples were tested using two-tailed Student´s t-test. No significant difference ($P<0.05$) was observed.

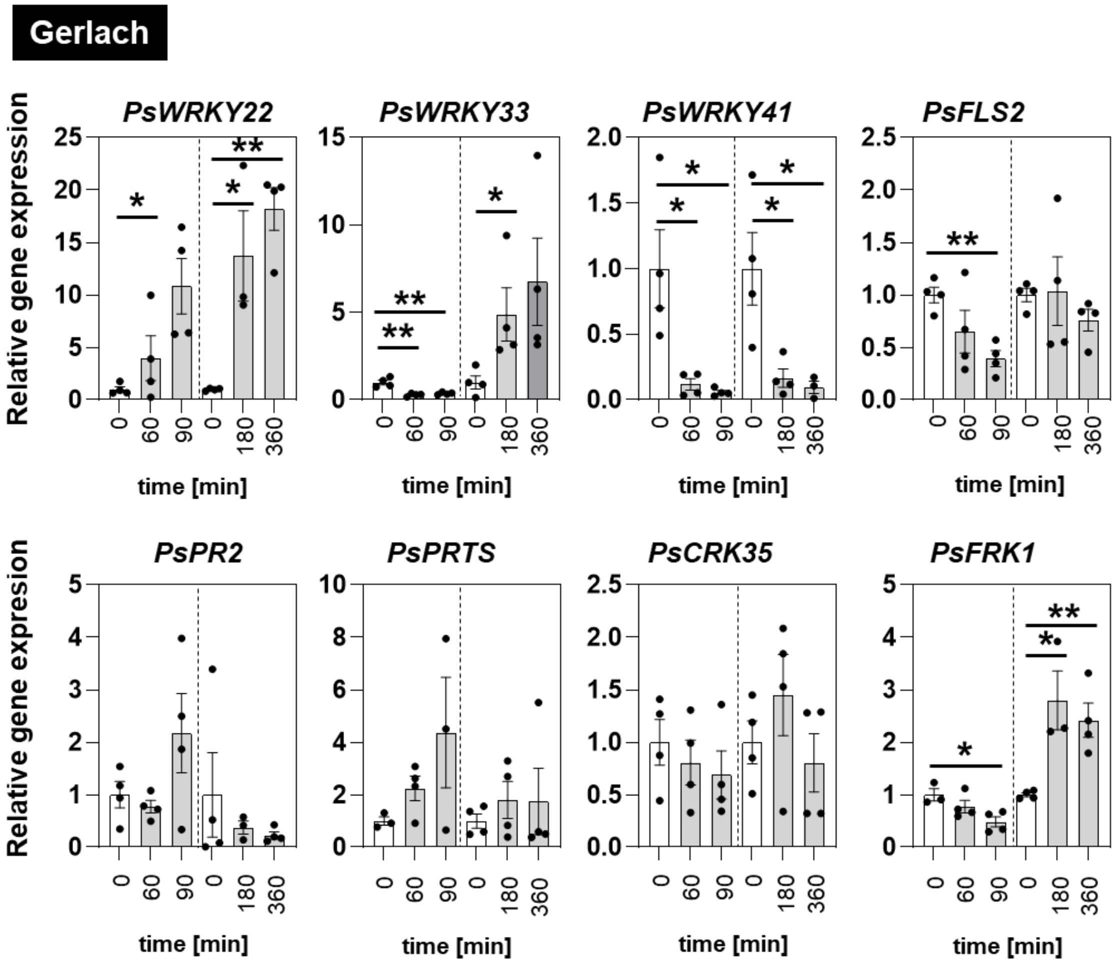

Figure S13. Gene expression after treatment with H_2O compared to non-treated samples. The leaf discs were cut from 5-6 week old poppy (cultivar Gerlach) and left overnight in darkness and after that treated with H_2O for 60, 90, 180, 360 minutes. Expression values were normalized to the control samples (no treated, directly harvested samples), which were set to 1. Expression levels in treated samples are presented relative to the corresponding control. The data represent the means + SEM; n=3-4. Asterisks indicate that the mean value is siginificantly different from the control conditions (two-tailed Student´s t-test, *P<0.05, **P<0.01).

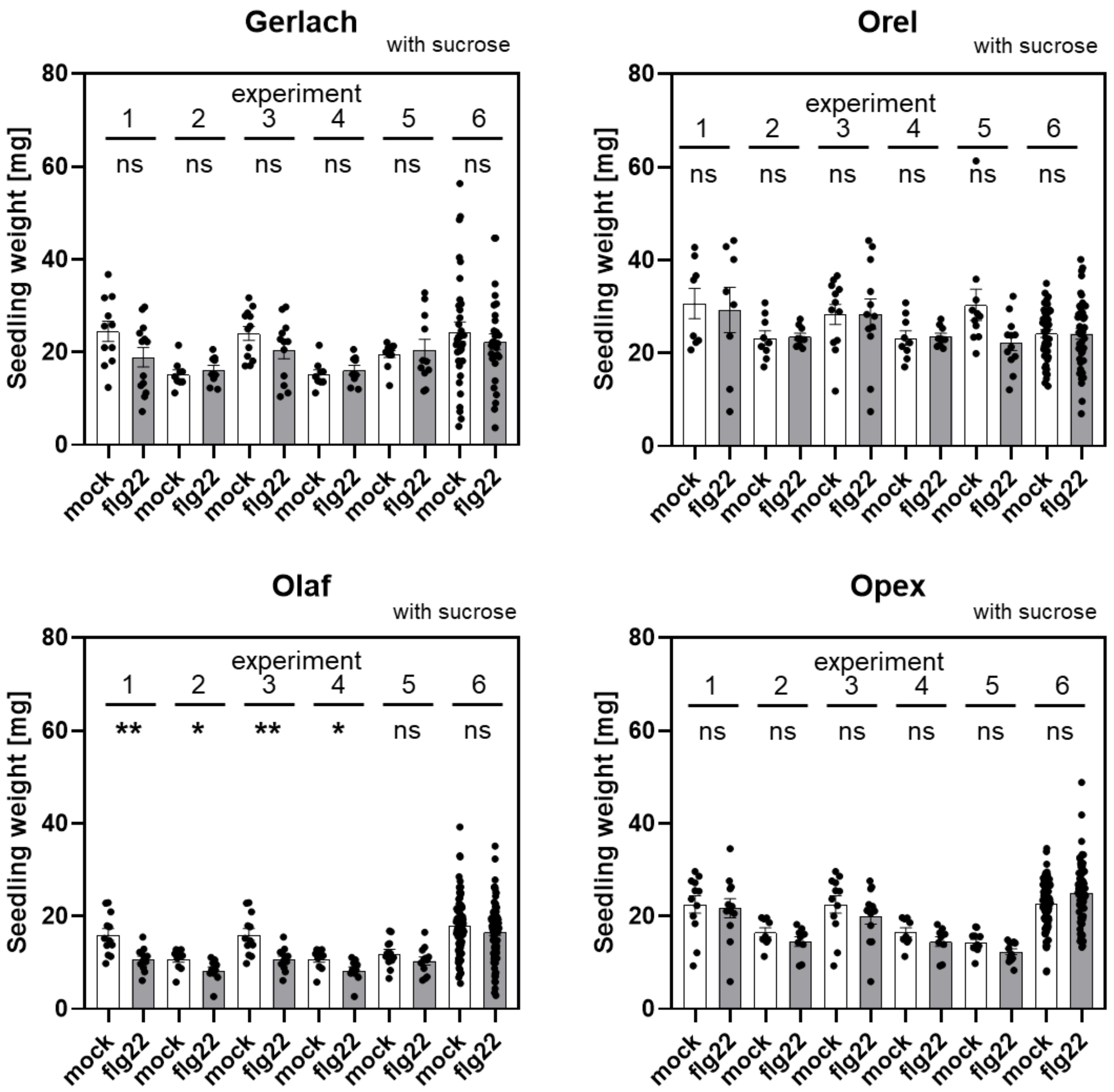

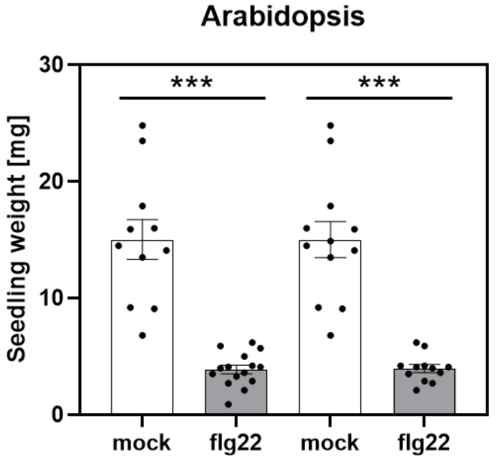

Figure S14. Raw data for seedlings growth inhibition in media containing sucrose shown in figure 5. The values represent mean with SEM, n=8-24. Asterisks indicate that the mean value is siginificantly different from the respektive control conditions (two-tailed Student´s t-test, *P<0.05, **P<0.01, ***P<0.001).

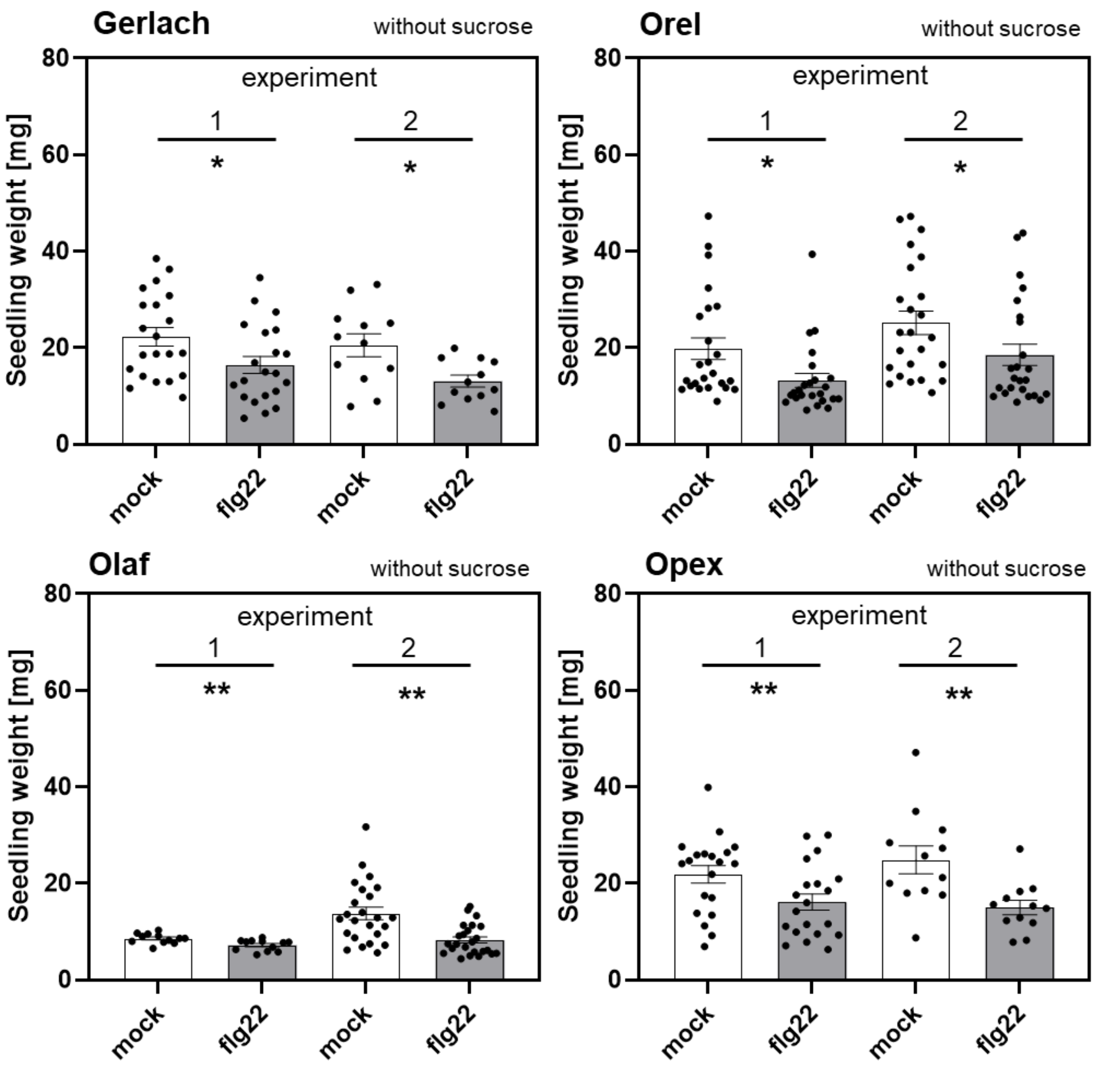

Figure S15. Raw data for seedlings growth inhibition in media without sucrose shown in figure 5. The values represent mean with SEM, n=12-24. Asterisks indicate that the mean value is siginificantly different from the control conditions (two-tailed Student´s t-test, *P<0.05, **P<0.01).

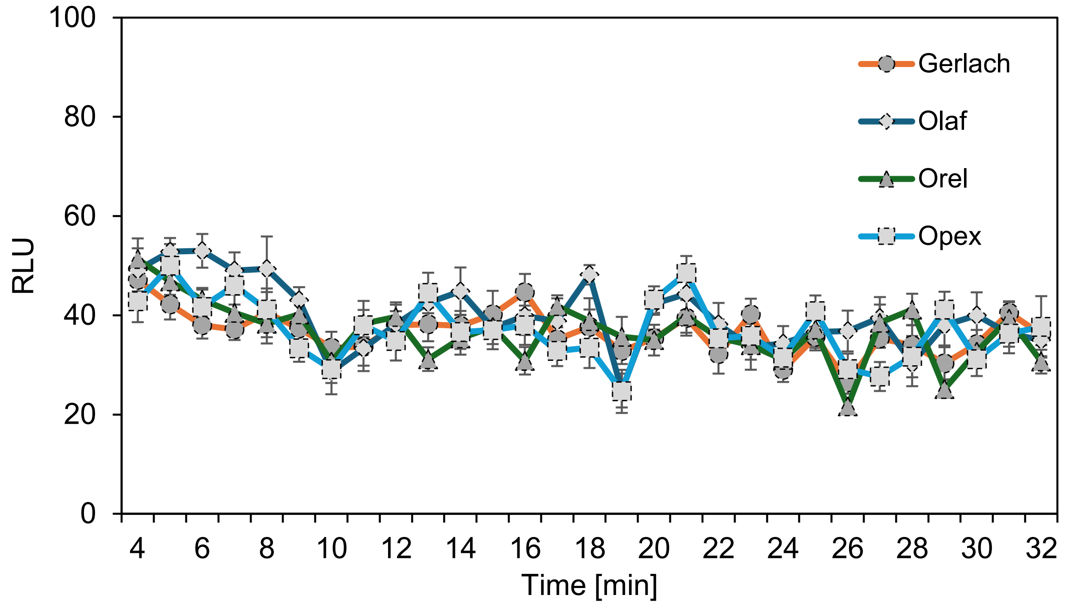

Figure S16. ROS burst in seedlings of four poppy cultivars after flg22 treatment. 7-day old seedlings were treated with 5µM flg22. The data represent the means and SEM; n=12 seedlings. The experiment was done in three biological repeats with similar results.

Table S1. Primers used for RT-qPCR in *Papaver somniferum*

Accession (NCBI)	(putative) Gene name	Primer name	Orientation	Sequence	Product lenght	Reference	Notes
LOC113344176	*PsWRKY22*	qPCR_PsWRKY22_FP	Forward	TCCAAGAGGATATTACAGATGC	124	this study	WRKY transcription factor 22
		qPCR_PsWRKY22_RP	Reverse	AACGGCATGATTATGTTCAG		this study	
LOC113329236	*PsWRKY33*	qPCR_PsWRKY33_FP	Forward	CTCTTCCTCCAATATTCTTCCA	122	this study	Probable WRKY transcrioption factor 33
		qPCR_PsWRKY33_RP	Reverse	CTGTTTCTTGCCTGATTGTG		this study	
LOC113321464	*PsWRKY41*	qPCR_PsWRKY53_FP	Forward	GGGTCTCCGTCGATTCGTTGA	130	Ray et al. 2021	In Ray et. al. named according to soybean as probable WRKY53.
		qPCR_PsWRKY53_RP	Reverse	ACAGCTGGAGAAAGTACGGGC		Ray et al. 2021	
LOC113355437	*PsFLS2*	qPCR_PsFLS2_FP	Forward	CTCAACACTGAGCTCCTACG	113	this study	LRR receptor-like serine/threonine protein kinase
		qPCR_PsFLS2_RP	Reverse	TCCCTGCATAGTTGTGTCC		this study	
LOC113274805	*PsPR2*	qPCR_PsPR2_FP	Forward	AAATGTATTCCTTGAAGCGGC	158	this study	Putative glucan endo-1,3-beta-glucosidase
		qPCR_PsPR2_RP	Reverse	CTCGTAGGGCATTTAGGGC		this study	
LOC113282088	*PsPRTS*	qPCR_PsPRTS_FP	Forward	TGACGACCCCAGAATCTCCC	113	Ray et al. 2021	In Ray et al. named according soybean genome as Pathogenesis-related thaumatin superfamily
		qPCR_PsPRTS_RP	Reverse	CAGCCAAGTGCAGGGAAACC		Ray et al. 2021	
LOC113329942	*PsCRK35*	qPCR_PsCRK1_FP	Forward	TGGCGAAGGTGGATTTGGAT	107	Ray et al. 2021	In Ray et al. named according soybean genome as Cysteine-rich RLK isoform 1
		qPCR_PsCRK1_RP	Reverse	ACTCTTGTTCGCCTTGTCCA		Ray et al. 2021	
LOC113321890	*PsFRK1*	qPCR_PsFRK1_FP	Forward	CGTGGGTAAACTGTAACTCGG	135	this study	Probable LRR receptor-like serine/threonine protein kinase
		qPCR_PsFRK1_RP	Reverse	AATTCCCGCCAAGTCTTAGC		this study	
LOC113323526	*Actin*	qPCR_Actin_FP	Forward	GCTGTCCTTTCCCTCTACGC	155	Zhang et al. 2020	Actin-11
		qPCR_Actin_RP	Reverse	AGGGCATCAGTAAGGTCACG		Zhang et al. 2020	

Table 2. List of binding affinity energies for *P. somniferum* and *A. thaliana* FLS2 and flg22 and *Xcc*Flg22

# model	Ligand	Binding Affinity (kcal/mol)	*in silico* Kd (µM)
0	*Ps*FLS2+*Xcc*Flg22	-7.4	3.605
1	*Ps*FLS2+*Xcc*Flg22	-7.2	5.058
2	*Ps*FLS2+*Xcc*Flg22	-7.2	5.058
3	*Ps*FLS2+*Xcc*Flg22	-7.1	5.992
4	*Ps*FLS2+*Xcc*Flg22	-6.9	8.408
5	*Ps*FLS2+*Xcc*Flg22	-6.9	8.408
6	*Ps*FLS2+*Xcc*Flg22	-6.9	8.408
7	*Ps*FLS2+*Xcc*Flg22	-6.8	9.960
8	*Ps*FLS2+*Xcc*Flg22	-6.8	9.960
0	*Ps*FLS2+flg22	-6.5	16.555
1	*Ps*FLS2+flg22	-6.3	23.230
2	*Ps*FLS2+flg22	-6.2	27.517
3	*Ps*FLS2+flg22	-6.1	32.595
4	*Ps*FLS2+flg22	-6.1	32.595
5	*Ps*FLS2+flg22	-6.1	32.595
6	*Ps*FLS2+flg22	-6	38.611
7	*Ps*FLS2+flg22	-6	38.611
8	*Ps*FLS2+flg22	-6	38.611
0	*At*FLS2+flg22	-13.4	0.000139
1	*At*FLS2+flg22	-12.9	0.000325
2	*At*FLS2+flg22	-12.7	0.000456
3	*At*FLS2+flg22	-12.4	0.000757
4	*At*FLS2+flg22	-12.4	0.000757
5	*At*FLS2+flg22	-12.3	0.000897
6	*At*FLS2+flg22	-12	0.001491
7	*At*FLS2+flg22	-11.9	0.001766
8	*At*FLS2+flg22	-11.9	0.001766
